# Drebrin Upregulation Regulates Astrocyte Polarization and Supports Tissue Recovery After Spinal Cord Injury in Mice

**DOI:** 10.1002/glia.70048

**Published:** 2025-06-11

**Authors:** Barbora Smejkalová, Marta Ornaghi, Kateřina Štěpánková, Juliane Schiweck, Lucia Machová Urdzíková, Robert Huelse, Susanne Mueller, Philipp Boehm‐Sturm, Jessica C. F. Kwok, James Fawcett, Kai Murk, Britta J. Eickholt, Pavla Jendelová

**Affiliations:** ^1^ Institute of Experimental Medicine, Czech Academy of Sciences Prague Czech Republic; ^2^ Second Faculty of Medicine Charles University Prague Czech Republic; ^3^ Institute of Biochemistry, Charité Universitätsmedizin Berlin Berlin Germany; ^4^ Freie Universität Berlin Berlin Germany; ^5^ German Center for Neurodegenerative Diseases (DZNE) Bonn Germany; ^6^ Charité‐Universitätsmedizin Berlin, Charité Core Facility Experimental MRIs Berlin Germany; ^7^ Faculty of Biological Sciences University of Leeds Leeds UK; ^8^ Department of Clinical Neurosciences John Van Geest Centre for Brain Repair, University of Cambridge Cambridge UK; ^9^ Charité ‐ Universitätsmedizin Berlin, Institute of Molecular Biology and Biochemistry Berlin Germany

**Keywords:** immune cell infiltration, neurodegeneration, reactive astrogliosis, spinal cord injury

## Abstract

Spinal cord injury (SCI) results in significant disruption of nerve fibers responsible for transmitting signals between the brain and body, often leading to partial or complete motor, sensory, and autonomic dysfunction below the injury site. Astrocytes are an important component in scar formation, crucial for suppression of injury propagation, effective wound healing, and the regulation of neuronal plasticity. Here, we identify the role of the actin‐binding protein Drebrin (DBN) in reactive astrogliosis following SCI. SCI induces the upregulation of DBN in astrocytes, which controls immediate injury containment but also the long‐term preservation of tissue integrity and healing in the spinal cord. DBN knockout results in enlarged spinal cord lesions, increased immune cell infiltration, and neurodegeneration. Mechanistically, DBN loss disrupts the polarization of scar border‐forming astrocytes, leading to impaired encapsulation of the injury. In summary, DBN serves as a pivotal regulator of SCI outcome by modulating astrocytic polarity, which is essential for establishing a protective barrier confining the lesion site.

## Main Points

- Absence of DBN leads to larger spinal lesions, immune cell infiltration and attenuated astrocyte reactivity in the early phase post SCI.
- Absence of DBN leads to greater neurodegeneration, underscoring its importance in tissue repair.

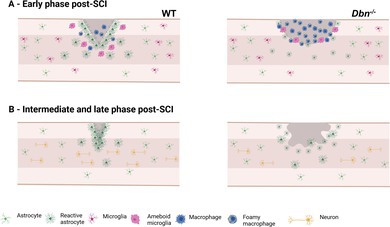

## 1 Introduction

Injury to the central nervous system (CNS), such as spinal cord injury (SCI), leads to severe neurological deficits due to irrevocable neuronal cell death, inflammation, and a failure in axonal regeneration and reconnection (Ahuja et al. 2017). Accordingly, the consequences of SCI are usually a partial or complete loss of motor function, sensation, and autonomic control below the level of the injury (Ahuja et al. 2017). Effective therapeutic strategies to promote neural repair and functional recovery are hindered by the complex cellular and molecular responses to CNS injury.

Major hallmarks of penetrating CNS injuries are the formation of dense boundaries in and around lesion cores by non‐neuronal cells. In SCI, fibroblasts, pericytes, and other mesenchymal cells secrete extracellular matrix (ECM) dense fibrotic tissue in lesion cores. The fibrotic area is surrounded by a scar‐barrier, the main components of which are microglia and reactive astrocytes (Bradbury and Burnside 2019). The scar‐barrier formation evokes substantial changes in astrocyte function and morphology. Injury signals lead to hypertrophy of astrocyte soma and main protrusions, while reactive astrocytes proximal to lesion sites polarize and extend long palisade‐like processes (Schiweck et al. 2018). It is widely accepted that reactive astrocytes fulfill, in these settings, important beneficial roles, as they participate in the containment of local pathological incidents by restricting inflammation and shielding neurons, maintaining neuronal survival and functionality, and re‐sealing the blood–brain barrier (BBB) (Burda et al. 2016; Schiweck et al. 2018). Reactive astrocytes, therefore, provide critical neuroprotection by protecting the surrounding uninjured CNS tissue. However, it is currently debated whether reactive astrocytes impair or promote regenerative axon growth (Anderson et al. 2016). Astrocytes and astrocyte‐secreted molecules, such as CSPGs, contribute to the physical and chemical barrier that obstructs axon regrowth (Bradbury and Burnside 2019). This complex interplay of astrocytes with neurons and non‐neuronal cells is a subject of intensive research. However, the molecular mechanisms controlling astrocyte reactivity, such as cytoskeleton remodeling and membrane trafficking processes, are largely unknown.

Previously, we demonstrated the impact of the actin binding protein Drebrin (DBN) on astrocyte reactivity and neuroprotection in traumatic brain injury (TBI). As an actin filament‐stabilizing protein widely expressed in neurons throughout the CNS, DBN protects the structural integrity of dendritic spines from oxidative stress (Aoki et al. 2005; Kreis et al. 2019). DBN is highly expressed in neurons, but is practically non‐detectable in brain astrocytes under steady‐state conditions. However, mild brain stab injuries trigger an immediate upregulation of DBN in astrocytes, where it is key for the dynamic organization of the actin cytoskeleton. The DBN‐dependent switch in actin networks enables reactive astrocytes to establish distinct membrane trafficking routes for the re‐distribution of surface receptor and adhesion molecules such as β1‐integrin. Our analyses of TBI in *Dbn* ^*−/−*^ mice showed further that this DBN‐dependent mechanism is integral for reactive astrogliosis and neuroprotection. In this experimental paradigm, DBN loss causes prominent defects in astrocyte reactivity and boundary formation, followed by abnormally excessive neurodegeneration (Schiweck et al. 2021). The substantial exacerbation of minor brain injuries in DBN deficient mice prompted us to ask whether DBN acts in a similar manner in different CNS injury models.

To test this hypothesis, we performed thoracic SCIs in our established DBN knockout mouse model (*Dbn* ^*−/−*^) (Willmes et al. 2017). Following SCI, the inflicted tissue damage is far greater than in our previously exploited brain injury model (Schiweck et al. 2021), causing white matter deterioration in addition to a more rapid and severe immune response. As a major trigger of the immune response, SCI induces the invasion of both microglia and peripheral macrophages (Hellenbrand et al. 2021). We followed the SCI pathophysiological impact over several weeks in *Dbn* ^*−/−*^ and WT mice and found perturbed astrocyte boundaries, larger lesion sites, and aggravated neurodegeneration in *Dbn* ^*−/−*^ mice when compared to WT. These hallmarks were associated with an increased number of microglia and macrophages at SCI sites. Our findings demonstrate that DBN plays a prominent role in SCI during the initial phase of damage containment, as well as in long‐term injury maintenance and repair processes.

## 2 Materials and Methods

### 2.1 Experimental Animals

All procedures were performed in accordance with the relevant guidelines and regulations. Animal procedures were approved by the ethical committee of the Institute of Experimental Medicine of the Academy of Science of the Czech Republic (ASCR) and performed in accordance with Law No. 77/2004 of the Czech Republic (Ethics approval number: 13/2020; Project number: AVCR 7848/2022 SOV II). All work was performed according to the European Commission Directive 2010/63/EU and the ARRIVE guidelines. Every effort was made to minimize pain and suffering. Animals were kept in individually ventilated cages in standard conditions, under a 12:12 h light/dark cycle with free access to water and food pellets.

A total of 67 adult, male and female, mice of *C57B/6* or *Dbn* ^*−/−*^ genetic background (Willmes et al. 2017) were used. Out of these, 17 animals (7 male, 10 female), either *Dbn* ^*−/−*^ (*n* = 8) or their WT (*n* = 9) littermates, were used for behavioral testing.

### 2.2 Animal Procedures

Animals underwent a thoracic SCI, dorsal column crush (DCC), at the Th8 segment of the spinal cord. The mice were induced with 5% (v/v) Isoflurane and maintained on 1.0% Isoflurane in 1.0 L/min air during the surgery. Animals were injected subcutaneously with buprenorphine (Bupaq; 0.05 mg/kg body weight) for analgesia. Surgeries were performed under aseptic conditions. An incision was made from level Th6 to Th12 of the spine and laminectomy of the Th8 dorsal vertebral arch was performed. The spinal cord dorsal column tract and CST were crushed with Bonn micro forceps (FST, No. 11083–07); the dimensions of the injury were approximately 1 mm width and 0.8 mm depth. The animals received post‐operative care. The experimental animals were divided into different time‐point groups: an early (1–2 weeks after SCI), an intermediate (4 weeks after SCI), and a late (8 weeks after SCI) group.

At the end of the experiments, the mice were induced with 5% (v/v) Isoflurane and then intraperitoneally anesthetized with a lethal dose of ketamine (100 mg/kg) and xylazine (20 mg/kg), perfused intracardially initially with PHEM cytoskeleton preservation buffer (25 mM HEPES, 60 mM PIPES, 10 mM EGTA, 2 mM MgCl_2_, pH 7.4) followed by 4% (w/v) paraformaldehyde in PHEM buffer (PFA‐PHEM) and post‐fixed in the same solution for 24 h. Spinal cords were dissected and treated with 10%, 20%, and 30% (w/v) sucrose (Millipore, 107,651) in PHEM before embedding into OCT mounting medium (VWR, #03820168) and sectioning on a cryostat to 20 μm sections.

The anesthesia used has previously been shown not to interfere with animal behavior.

### 2.3 Ex Vivo MRI

Magnetic resonance imaging (MRI) was performed at a 7 Tesla rodent scanner (BioSpec 70/20 USR, Bruker, Ettlingen, Germany) running Paravision 6.0.1 software with a mouse head transmit/receive ^1^H‐CryoProbe (Bruker). The spinal cord samples were fixed in 4% paraformaldehyde for a minimum of 24 h and then rehydrated in 1x phosphate‐buffered saline. To avoid signal from background, the samples were placed in 5 mm NMR tubes filled with hydrogen‐free perfluoropolyether oil (FomblinY, Sigma Aldrich). For high‐resolution morphological imaging, a T_2_*‐weighted 3D sequence was used (FLASH) with a field of view (FOV) = 12 × 18 × 9 mm^3^ and a matrix size (MTX) = 300 × 450 × 225, resulting in an isotropic spatial resolution of (40 μm)^3^. Further imaging parameters were repetition time (TR) = 36 ms, echo time (TE) = 5.6 ms, flip angle = 20°, readout bandwidth (BW) = 50 kHz, number of averages (NA) = 15, resulting in a total scan time (TA) = 10:07 h. Volumes of interest (lesion core and the extended lesion) were manually segmented on FLASH images in Analyze 10.0 (AnalyzeDirect Inc., Overland Park, USA) 3D visualizations were generated in MRIcroGL (https://www.nitrc.org/projects/mricrogl).

### 2.4 Behavioral Testing

The experimental animals underwent daily pre‐test sessions for 1 week prior to SCI. After the recovery period, they were tested at two, four, six, and eight weeks post‐SCI. The behavioral tests used included Basso Mouse Scale (BMS) walking score to study open field locomotion in spinal cord injured mice, maximum speed with rotarod to test motor coordination and balance, and ladder rung walking test to assess both skilled motor function and sensorimotor integration. For BMS, animals were observed and recorded for 4 min in the open field test area. The locomotor performance was then scored from the videos using the BMS scoring scale (Basso et al. 2006). The maximum rotarod speed test started at 4 rpm for 10 s; then the speed was accelerated by 20 rpm/min up to a maximum speed of 40 rpm. The last speed before the mouse fell off was recorded (Deacon 2013). The ladder rung walking test was performed as described by Metz and Whishaw (Metz and Whishaw 2002). Each animal was recorded while performing the task. Skilled locomotor ability was then assessed from the videos. Each step cycle was scored using the Metz‐Whishaw scoring scale and averaged. Males and females were tested separately. The experimenters were blinded to the groups during the whole course of behavioral testing and analysis. The genotype of the animals was revealed only after the evaluation.

### 2.5 Tissue Preparation and Immunohistochemical Staining

Approximately 1.5 cm‐long pieces of spinal cords with the Th8 level in the middle were embedded in OCT Embedding matrix (VWR, 361603E), frozen to −20°C, and serially sectioned on a cryostat into 20 μm‐thick sagittal sections for immunohistological analysis. Tissue sections were mounted on Superfrost Plus Gold slides (VWR, 630–1324) and stored at −20°C prior to immunohistological staining.

The slides were washed with PHEM and permeabilized with 0.5% (v/v) Triton X‐100 in 1X PHEM for 30 min. Tissue was then blocked with ChemiBLOCKER (1:10; Millipore, 2170), 0.3 M glycine, 0.2% (v/v) Triton X‐100 in 1X PHEM for 2 h. The sections were then incubated with the following primary antibodies: mouse anti‐Arg1 (OriGene Technologies Inc., UM800178, 1:500), rabbit anti‐DBN (Boster Biological Technology, Pleasanton CA, USA, M05530, 1:200), guinea pig anti‐GFAP (Synaptic Systems, 173,004), chicken anti‐GFAP (Abcam, ab4674‐50 μL, 1:1000), mouse anti‐GFAP (Cy3 conjugate, Sigma‐Aldrich, C9205), chicken anti‐IBA1 (Synaptic Systems, 234,009, IHC 1:500), rabbit anti‐iNOS (Invitrogen, PA1‐036, 1:500), rabbit anti‐MAG (Cell Signaling, 9043S, 1:200), and rabbit anti‐Synaptophysin (Abcam ab32127, 1:500) for 48 h at 4°C, washed, and labeled with fluorescent‐conjugated secondary antibodies (1:400, 4 h, room temperature).

Cross‐absorbed secondary antibodies conjugated to cyanine or Alexa dyes were purchased from Dianova or Invitrogen: donkey anti‐guinea Pig IgG (H + L)‐Cy3 (Dianova,706–165‐148), donkey anti‐rabbit IgG (H + L)‐Alexa Fluor 647 (Dianova, 711–605‐152), donkey anti‐rabbit IgG (H + L)‐Alexa Fluor 488 (Dianova, 711–545‐152), donkey anti‐chicken IgY (H + L)‐Alexa Fluor 647 (Dianova, 703–605‐155), goat anti‐chicken IgY (H + L)‐Alexa Fluor 488 (Invitrogen, A‐11039), goat anti‐chicken IgY (H + L)‐Alexa Fluor 633 (Invitrogen, A‐21103), goat anti‐mouse IgG (H + L)‐Alexa Fluor 488 (Invitrogen, A‐11001), goat anti‐rabbit IgG (H + L)‐Alexa Fluor 488 (Invitrogen, A‐11012). DNA staining was carried out using Hoechst 33258 (Thermo Scientific, H3569, 1:10,000).

### 2.6 Histology

For Oil Red O staining, spinal cord sections (mounted on Superfrost Plus Gold slides) were rinsed with 60% isopropyl alcohol (Penta, 17,510), stained for 15 min with Oil Red O working solution (Sigma‐Aldrich, O0625) and differentiated in 60% isopropyl alcohol for 2 min. Then the slices were stained in hematoxylin (Sigma‐Aldrich, H3136) solution for 2 min, rinsed twice with distilled H_2_O, and mounted in Mowiol mounting medium.

For Nissl body analysis, spinal sections (mounted on Superfrost Plus Gold slides) were preincubated in a descending ethanol series (96%, 90%, 70%, 50% and 30% ethanol, each step for 2 min). Cresyl violet (acetate) (Sigma‐Aldrich, 1.05235) working solution was freshly prepared in the manufacturer's acetate buffer solution (pH 3.6) and filtered prior to use. Then, sections were stained in cresyl violet solution for 20 min. Subsequently, sections were incubated for 1 s in 96% ethanol and washed briefly with 70% ethanol. Clearing of sections was performed by two subsequent incubations in xylene for 2 min. Stained sections were mounted in water‐free DPX medium (Sigma‐Aldrich).

For the myelin staining with Luxol Fast Blue, slides with spinal cord sections were incubated overnight at 60°C in the Luxol Fast Blue solution 10 g Solvent Blue 38 (Sigma, S3382), 950 mL 96% ethanol (Penta, 70,390, 50 mL 99% acetic acid (Penta, 20,000)). The slides were briefly washed with 70% ethanol (Penta, 70,392) and differentiated with a lithium carbonate solution (Sigma, 203,629, 0, 5% solution in dH_2_O). Sections were dehydrated in 96% and 100% ethanol, cleared with xylene (Penta, 28,440) and mounted in Solakryl BMX (26,210, Penta).

### 2.7 Microscopy and Statistical Analysis

Staining was imaged using a LEICA CTR 6500 microscope with FAXS 4.2.6245.1020 (TissueGnostics, Vienna, AT) software (brightfield images), Olympus SpinSR Yokogawa CSU‐W1 with cellSens (Ver. 3.2) imaging software, Nikon Spinning Disk Confocal CSU‐W1 SoRa or Nikon A1Rsi + confocal microscope, Zeiss LSM 880 Airyscan with ZEISS ZEN Black Edition software.

Microscopy images were processed and analyzed in FiJi (Schindelin et al. 2012) and/or Imaris (Oxford Instruments, version 9.7.2). For intensity measurements, mean gray values of areas of interest were measured in FiJi after subtracting the background signal, which was obtained from outside the samples. For coverage measurements, thresholds were applied in FiJi to the images to determine the area fraction with positive immunoreactivity. To highlight and measure DBN immunoreactivity in GFAP+ astrocytes and IBA1+ microglia, we analyzed DBN immunoreactivity relative to signals of the respective marker proteins using the CoLoc application in Imaris. DBN signal quantifications were conducted within masks generated from GFAP or IBA1 immunoreactivity, respectively.

For analysis of astrocyte processes, Z‐stacks of GFAP signal were used to create a reconstruction in Imaris; an astrocytic net representation was created using the Filament tracing tool. Statistical analysis of process orientation angle and process mean diameter was exported from the software. *N* = 3 animals per group, *n* = 27 cells per group. To analyze astrocyte morphology in uninjured tissue, we first used the Filament tracing tool in Imaris. Processes were defined by GFAP immunoreactivity. The number of intersections per radius was obtained by performing Sholl analysis. *N* = 3 animals per group, number of cells analyzed *n* = 20 for WT and *n* = 21 for *Dbn* ^*−/−*^.

Image processing techniques for quantitative measurements, such as thresholding and background subtraction, were applied on individual channels in the same manner to entire images and across genotypes. To improve visibility, only images included in figures were subjected to linear adjustments in brightness and contrast using FiJi without obstructing or omitting details.

Statistical analysis was performed using GraphPad Prism software (9.0 version). Data are shown as mean ± SEM. Statistical differences between groups were determined by t‐test or two‐way ANOVA with Mann–Whitney test or Bonferroni‐Šídák's multiple comparison test. For the statistical evaluation of Sholl analysis, two‐way ANOVA with mixed‐effects model was used. The statistical tests used are mentioned in the legend below each figure. For all statistical analyses, a *p* value of 0.05 was considered significant (ns *p* ≥ 0.05, *p < 0.05, **p < 0.01, ***p < 0.001, **** *p* < 0.0001).

## 3 Results

### 3.1 DBN is Upregulated in Reactive Astrocytes After Thoracic SCI

**FIGURE 1:**
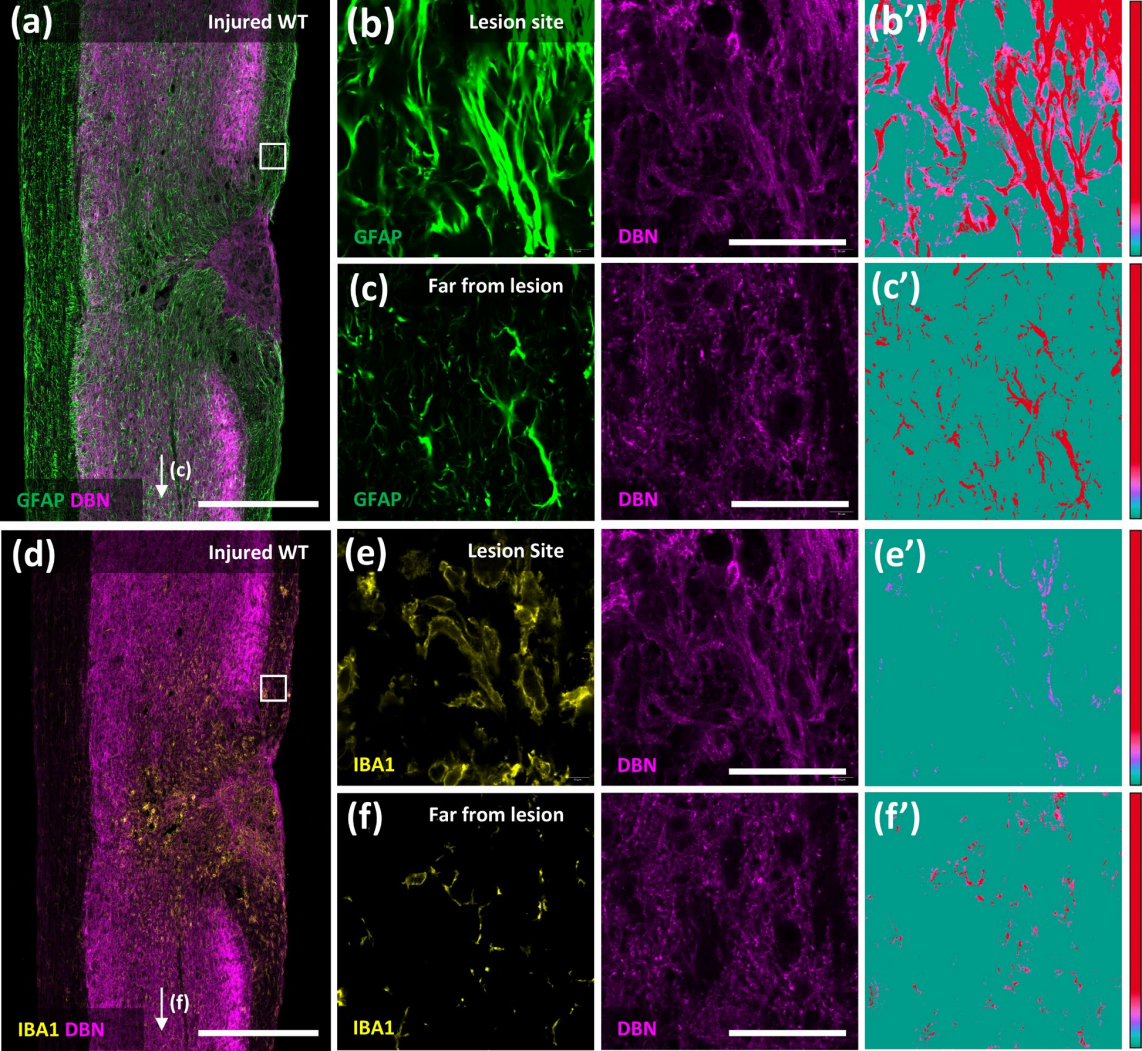
DBN is upregulated in astrocytes after thoracic spinal cord injury. Sections were triple‐stained with antibodies against DBN, GFAP and IBA1. (a) Overview of the lesion site in WT mice at 7 days post injury (early) timepoint labeled with anti‐GFAP and anti‐DBN antibodies, scale bar 500 μm. (b, c) close‐up images of the GFAP and DBN signal at the lesion site (b) and far from lesion site ((c), arrow in (a), 2500 μm far) respectively, scale bar 50 μm. (b’, c’)—Co‐localization analysis of GFAP and DBN immunoreactivity corresponding to (b) and (c), respectively. DBN co‐localization relative to GFAP is visualized as heatmap. (d) Overview of lesion site in WT mice 7 days post injury labeled with anti‐ DBN and anti‐IBA1 antibodies, scale bar 500 μm. (e, f) Close up images of the DBN and IBA1 immunoreactivity at the lesion site (e) and far from lesion site ((f) arrow in (d), 2500 μm far) respectively, scale bar 50 μm. (e’, f’) Co‐localization analysis of the DBN and IBA1 signals corresponding to (e) and (f), respectively. DBN co‐localization relative to IBA1 is visualized as heatmap. Note that panels (a–c) and (d–f) depict the same regions but are presented separately to allow distinct assessment of DBN co‐localization with either GFAP or IBA1.

To determine DBN protein levels in healthy and injured spinal cord of WT mice, we performed immunohistochemistry on spinal cord sections using DBN and glial fibrillary acidic protein (GFAP) antisera (Figures 1 and S1, S2). In uninjured tissue, DBN immunoreactivity was observed predominantly in the gray matter, where it was abundant in neuronal cell bodies and dendrites and at low level in GFAP‐positive astrocyte processes. Accordingly, DBN was not detected in white matter under physiological conditions (Figure S3). Following thoracic spinal cord injury, DBN was upregulated in GFAP‐positive astrocytic processes at injury sites, and in the lesion core (Figure 1a,b). We performed colocalization analyses using Imaris software. The colocalization index for GFAP and DBN at the lesion site was 31.99% (Figure 1b’). In contrast, astrocytes distant from lesion sites exhibit only minor increase in DBN expression; despite showing hallmarks of reactivity, such as hypertrophy (Figure 1c). The colocalization index shown only 4.16% colocalization of GFAP and DBN (Figure 1c’). In fact, astrocytes distant from the SCI lesion retained DBN levels and distribution in gray matter comparable to those in uninjured mice (Figure 1c–c’). We performed this colocalization analysis also in IBA1+ cells, another prominent subtype of cells responding in the early stages post SCI (Figure 1d–f’). We measured a very low colocalization index between IBA1 and DBN signal of only 1.52% at the injury site and 2.54% far from the lesion site. In addition, we performed the GFAP/DBN co‐localization analysis also at other timepoints post‐SCI. At 2 days post injury the GFAP/DBN co‐localization index was 6.67% in proximity to the lesion site (Figure S1a–c’). In this early SCI phase, astrocytes exhibit hypertrophy at this timepoint but have not polarized towards the lesion site yet. Ten days post‐injury, when the astrocytic barrier was in place, GFAP/DBN co‐localization was reduced to 20.55% (Figure S1d–f’). These findings indicate a transient peak in astrocytic DBN protein within the acute SCI phase at 7 days post injury.

We also analyzed the co‐localization of DBN with GFAP at 4 and 8 weeks post SCI, which correspond to intermediate and late SCI phases. At both points, we measured near the lesion sites low GFAP/DBN co‐localization indices of 6.91% in the intermediate and 4.82% in the late SCI phase, respectively (Figure S2). However, at these stages, a distinct DBN signal was observed outside of GFAP+ processes, possibly originating from the neuropil (Figure S2). Immunohistochemical analyses of DBN and GFAP in the uninjured spinal cord revealed a low co‐localization index of 3.41% (Figure S3a–d’). No DBN immunoreactivity was detected *in Dbn* ^*−/−*^ spinal sections highlighting the specificity of the DBN antibody (Figure S3b,e). In summary, these findings demonstrate that DBN is upregulated in astrocytes during the early stage of SCI. The DBN upregulation correlates with SCI phases when astrocytes undergo morphological changes to form a protective barrier.

### 3.2 Altered Astrocyte Activation and Injury Boundary Formation in *Dbn* ^*−/−*^ Mice Following SCI

**FIGURE 2:**
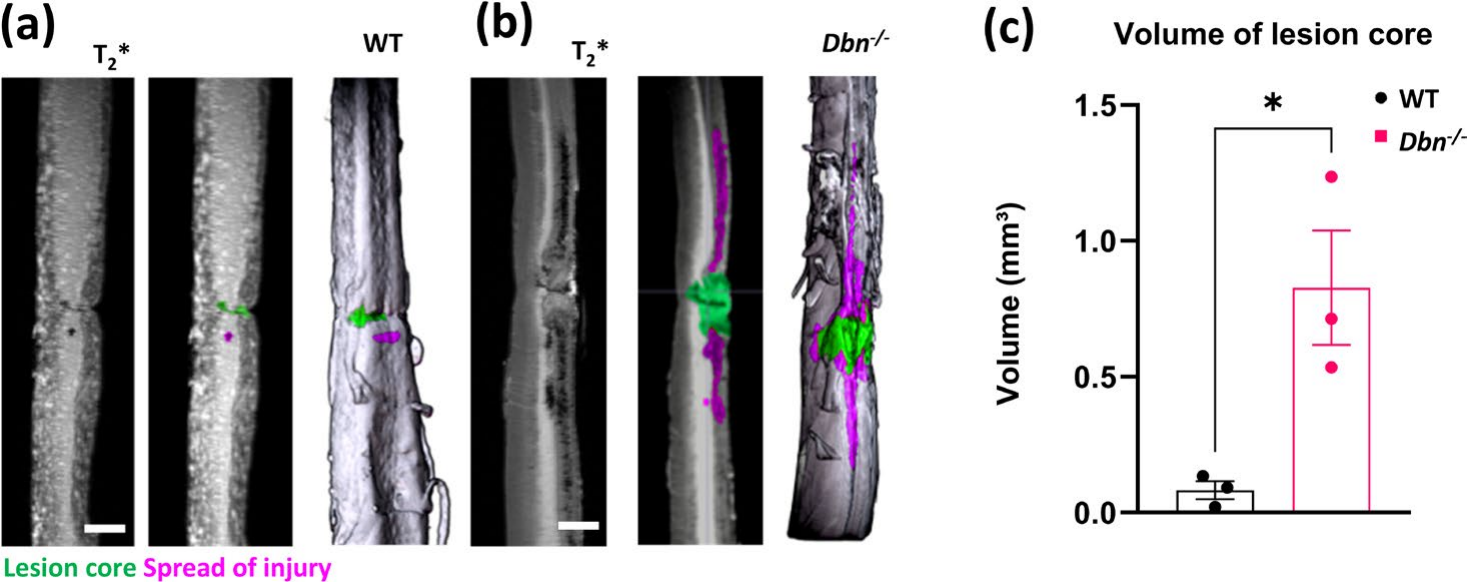
MRI of whole spinal cords 7 days post thoracic spinal cord injury—(a) T2*MRI image showing WT spinal cord 7 days post injury (left image), overlay of the image with highlighted lesion core (green) and injury spread area (mangenta, center image), and a corresponding 3D reconstruction with highlighted lesion core and injury spread (right image). Scale bar 1 mm. (b) MRI image showing a *Dbn* ^*−/−*^ spinal cord 7 days post injury (left image), overlay of the image with highlighted lesion core (green) and injury spread area (mangenta, center image), and a corresponding 3D reconstruction with highlighted lesion core and injury spread (right image). Scale bar 1 mm. (c) Quantification of the lesion volume from the 3D MRI reconstruction, data shown as mean ± SEM (*N* = 3), ns *p* ≥ 0.05, *p < 0.05, **p < 0.01, ***p < 0.001, ****p < 0.0001, t‐test, Two‐tailed.

**FIGURE 3:**
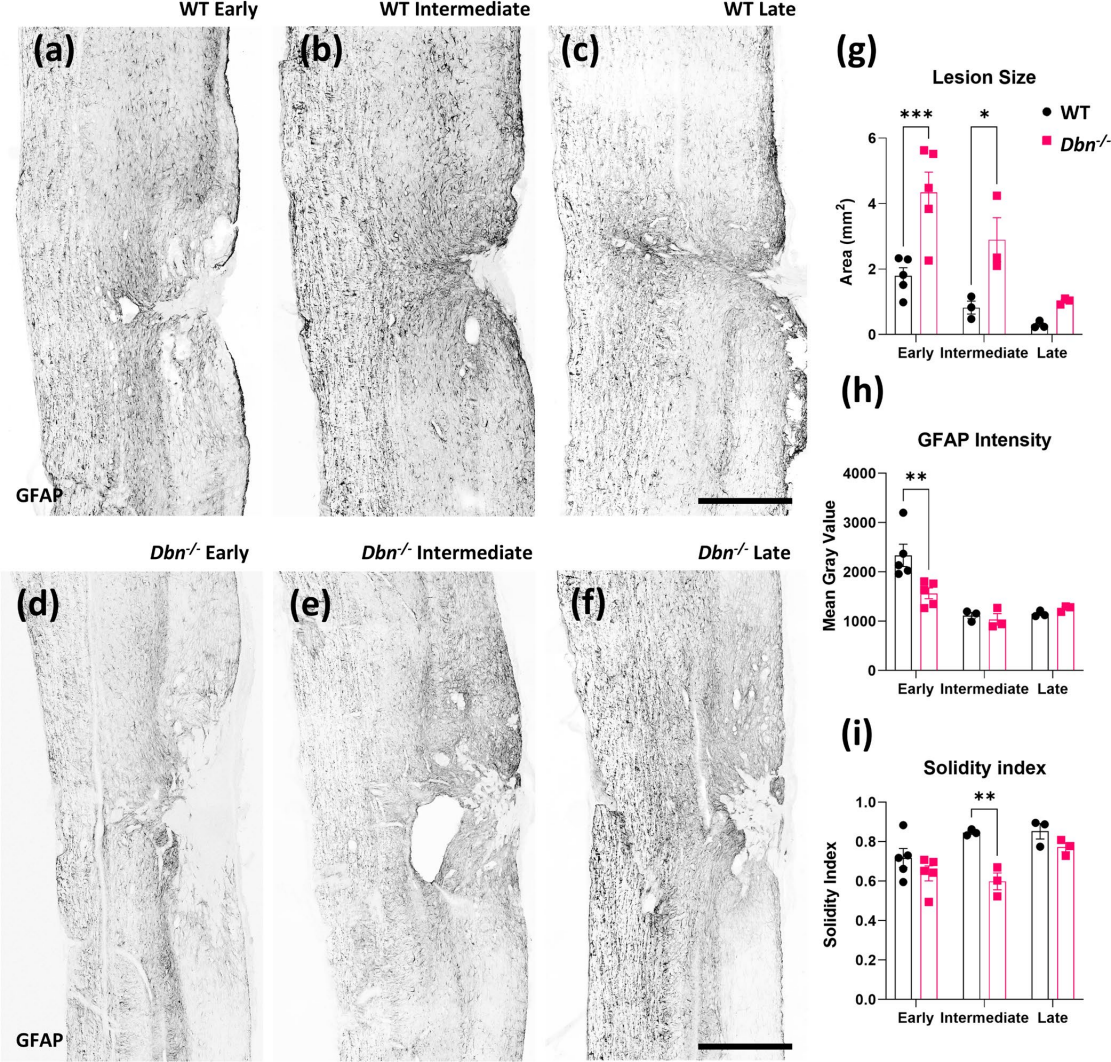
Altered astrocyte reactivity and scar formation following thoracic SCI in *Dbn* ^*−/−*^ mice—Representative images of GFAP immunoreactivity in WT tissue at (a) early timepoint (1–2 weeks), (b) an intermediate timepoint (4 weeks) and (c) a late timepoint (8 weeks) following SCI. Representative images of GFAP immunoreactivity in *Dbn* ^*−/−*^ tissue at (d) an early timepoint (1–2 weeks), (e) an intermediate timepoint (4 weeks), and (f) a late timepoint (8 weeks) following SCI, scale bars 500 μm. Graphs representing quantification of lesion size (g), GFAP intensity (h) and solidity index (i). Data shown as mean ± SEM, early timepoint (*N* = 5), intermediate and late timepoints (*N* = 3), ns *p* ≥ 0.05, *p < 0.05, **p < 0.01, ***p < 0.001, ****p < 0.0001, two‐way ANOVA, Bonferroni‐Šídák's multiple comparison test.

To investigate if DBN plays an integral role in SCI, we performed thoracic SCI using our established *Dbn* ^*−/−*^ mouse model, which we previously analyzed following mild traumatic brain injuries (Schiweck et al. 2021). We used MRI scans of whole extracted spinal cords to assess the overall lesion SCI size and parenchyma constitution at 7 days post‐injury. The MRI scans revealed larger lesion volumes in *Dbn* ^*−/−*^ mice when compared to WT. In the MRI, SCI sites in *Dbn* ^*−/−*^ mice exhibited areas with increased contrast in the dorsal ascending tracts also indicating a further spread of the injury (Figure 2). To study the SCI outcome in greater detail, we performed immunohistochemical analyses with high‐resolution confocal microscopy. We used GFAP immunohistochemistry to visualize the astrocyte boundaries. Thereby, we could assess the overall lesion sizes in early, intermediate, and late timepoints after SCI (Figure 3a–f), as well as the intensity of the GFAP signal as readout of astrocyte reactivity (Wanner et al. 2013). Our measurements of the lesion size revealed a significant increase in lesion area in *Dbn* ^*−/−*^ animals at early and intermediate timepoints, compared to lesions of corresponding WT mice (Figure 3g). The spreading of the injury area occurred primarily in a rostro‐caudal direction, with this pattern being most prominent at the early time point (Figure S4). Quantification of the GFAP signal in astrocytes adjacent to the lesioned area showed a significant decrease in signal intensity at the early timepoint after SCI in *Dbn* ^*−/−*^ mice when compared to WT mice (Figure 3h). However, at later timepoints, GFAP levels in *Dbn* ^*−/−*^ injury sites were not significantly different from those in WT control specimens. Along with the delay in GFAP upregulation, we observed differences in the shape of the lesion sites, which we quantified by morphometry using solidity index measurements (Figure 3i). In WT control animals, injury sites were initially irregularly shaped, but through constriction acquired increasingly regular morphologies in the intermediate SCI phase. In contrast, the tissue in *Dbn* ^*−/−*^ SCI appeared as ‘frayed’ throughout the initial and intermediate SCI phases due to incomplete scar‐barrier formation and microcavity development. These differences in wound size and shape became less prominent in the late SCI phase. Thus, our data indicate delays in wound constriction in *Dbn* ^*−/−*^ tissues, particularly in the intermediate phase of SCI. In summary, DBN deficiency attenuates GFAP upregulation and wound remodeling, particularly in the early and intermediate phases of SCI.

### 3.3 *Dbn* ^*−/−*^ Mice Show Decrease in Fine Motoric Skills in the Early Phase Post SCI

**FIGURE 4:**
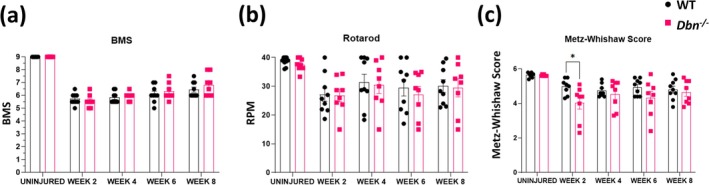
Behavioral analyses of WT and *Dbn* ^*−/−*^ mice following thoracic SCI—Results of motor tests of WT and *Dbn* ^*−/−*^ animals after SCI. Mice were pre‐tested before injury and then tested at 2, 4, 6, and 8 weeks post injury. (a) Basso Mouse Scale, (b) Rotarod maximum speed test, (c) Metz‐Whishaw score from ladder rung walking test (with unevenly spaced rungs). Data shown as mean ± SEM (*N* = 8–9 animals per group). ns *p* ≥ 0.05, *p < 0.05, **p < 0.01, ***p < 0.001, ****p < 0.0001, two‐way ANOVA, Bonferroni‐Šídák's multiple comparison test.

To test whether the absence of DBN and enlargement of spinal cord lesions would lead to greater motor impairment, we performed a set of behavioral tests on the hindlimb weekly for 8 weeks after SCI. The BMS score, which measures general locomotor function, was not significantly different between the *Dbn* ^*−/−*^ group and their WT littermates (Figure 4a). All tested animals scored lower 2 weeks after SCI, but both groups gradually improved over the course of testing, consistent with values observed in C57BL/6 animals (Basso et al. 2006). Similarly, no significant differences were observed in the maximum speed during the rotarod test, which evaluates balance and coordination (Figure 4b). The ladder walking test showed a significantly lower Metz‐Whishaw score in the *Dbn* ^*−/−*^ group compared to the WT 2 weeks after injury (Figure 4c). The Metz‐Whishaw score evaluates fine motor skills by scoring the precise placement of paws while walking on a ladder with unevenly spaced rungs. *Dbn* ^*−/−*^ animals displayed a greater frequency of slips and paw misplacements early after SCI but showed gradual improvement over the testing period, indicating partial recovery of motor coordination. These findings suggest that, although the absence of DBN leads to larger spinal lesions, it does not result in more severe gross motor skill impairment in mice.

### 3.4 Immune Cell Reactivity After SCI Differs in WT and *Dbn* ^*−/−*^ Tissue

**FIGURE 5:**
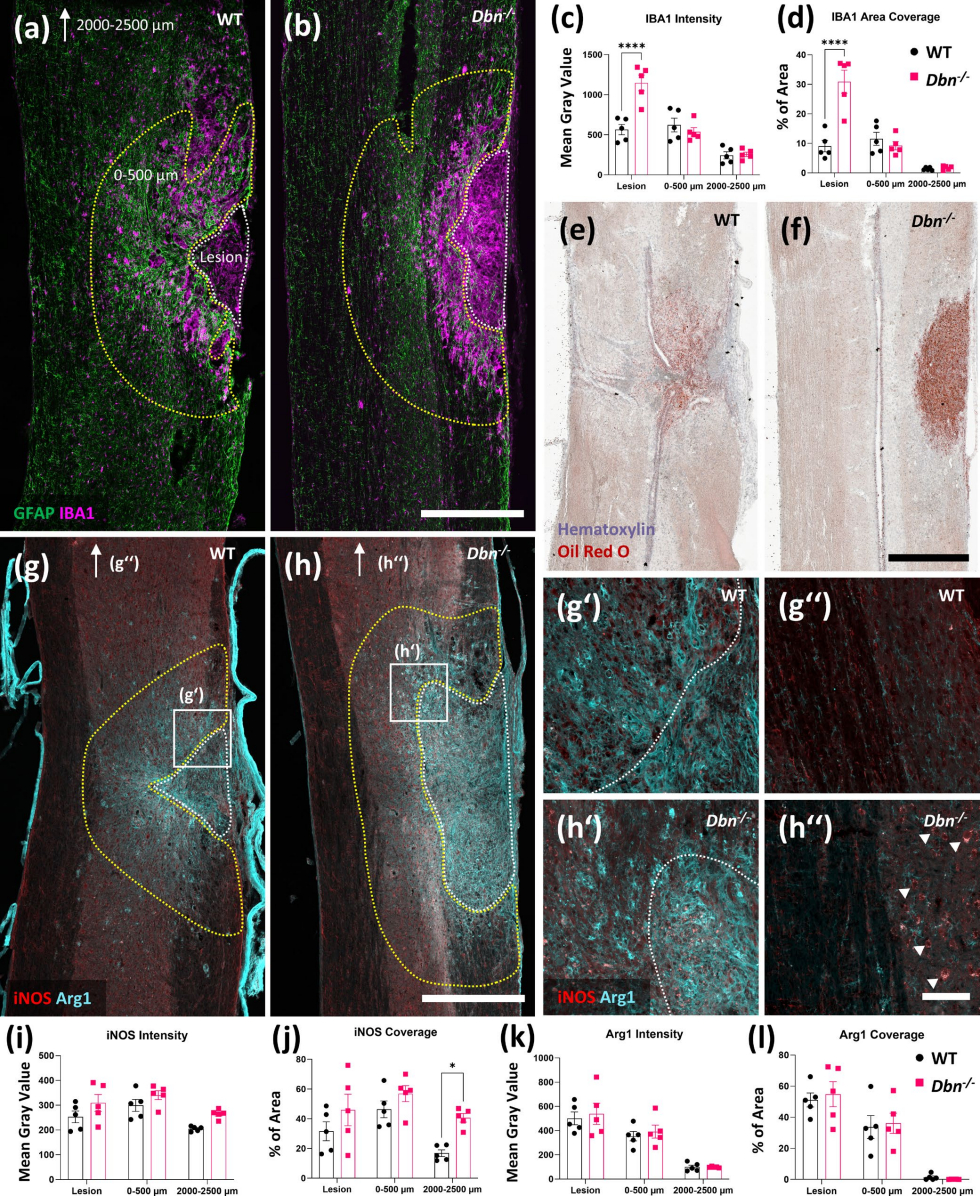
Immune cell reactivity after SCI differs in WT and *Dbn* ^*−/−*^ tissue—WT (a) and *Dbn* ^*−/−*^ (b) tissue was labeled at early timepoint with IBA1 and GFAP antibodies. Graphs show quantifications of IBA1 signal intensity (c) and area coverage (d). Oil Red O and hematoxylin staining in WT (e) and *Dbn* ^*−/−*^ (f) tissue. Analysis of Arg1 and iNOS signal in (g) WT and (h) *Dbn* ^*−/−*^ tissue, scale bar 500 μm. Details from WT tissue taken from lesion (g‘) and far from lesion (g”). Details from *Dbn* ^*−/−*^ tissue taken from lesion (h‘) and far from lesion (h”), scale bar 100 μm. White arrowheads show areas of increased iNOS positivity. Graphs show quantifications of iNOS (i, j) and Arg1 (k, l) intensity and area coverage, respectively. Measurements taken at lesion area (indicated with white dotted line), adjacent area 0–500 μm (indicated with yellow dotted line) and far from lesion (2000–2500 μm distant area of gray matter from the same slice, indicated by arrows in g and h, respectively). Data shown as mean ± SEM, (*N* = 5), ns *p* ≥ 0.05, *p < 0.05, **p < 0.01, ***p < 0.001, ****p < 0.0001, two‐way ANOVA, Bonferroni‐Šídák's multiple comparison test.

We then set out to analyze the immune response during the course of SCI in *Dbn* ^*−/−*^ and WT mice, including both reactive microglia and extravasating peripheral macrophages. We used IBA1 as a specific marker for microglia/macrophages and measured the IBA1 signal intensity and coverage in the lesion core and surrounding tissue in the early time point. IBA1‐positive cell numbers (area coverage) and IBA1 immunoreactivity were increased in the lesion core of *Dbn* ^*−/−*^ mice. However, we did not observe significant changes in IBA1 immunoreactivity in areas distant from the lesion core (Figure 5a–d). Macrophages play a key role in tissue debris removal following SCI injury via phagocytosis of myelinated fibers rich in lipids. The high lipid content and lipid metabolism dysregulation lead to their transformation into foamy macrophages, a phenotype associated with increased inflammation and potentially hampering nerve repair (X.‐X. Wang et al. 2024). Given the increase in IBA1+ cells accumulation in the lesion core, we used Oil Red O to stain neutral triglycerides and lipids deposited in foam cells. We observed a similar distribution to IBA1, indicating an increased deposition of foam cells in the spinal cord lesions of *Dbn* ^*−/−*^ mice (Figure 5e–f).

Microglia and macrophages can adopt various phenotypes depending on the stimuli they encounter; these are broadly classified as pro‐inflammatory (M1) or anti‐inflammatory (M2) (David and Kroner 2011). M1 phenotypes of microglia and macrophages exhibit increased expression of inducible nitric oxide synthase (iNOS), which we have accordingly used as an immunohistochemical marker for pro‐inflammatory cells, (Mantovani et al. 2004). M2 microglia and macrophages are distinguished by increased expression of arginase‐1 (Arg1) reflecting changes in their arginine metabolism. We used iNOS and Arg1 immunolabeling to study the abundance of M1 and M2 microglia/macrophages in WT and *Dbn* ^*−/*−^ mice after SCI. We observed no significant differences in M1 and M2 microglia/macrophages, neither in the lesion core nor in the surrounding tissue of WT and *Dbn* ^*−/−*^ mice (Figure 5g–i). However, we detected increased iNOS immunoreactivity in *Dbn* ^*−/−*^ tissue distant from the lesion site (Figure 5h). From the size, morphology, and distribution of the signal in the gray matter, we infer that this signal could originate from neuronal cells. We performed an analysis of these markers also in uninjured spinal cords, where we did not observe significant differences between both groups (Figure S5). In summary, we observed a higher abundance of IBA1+ cells in the lesion core of *Dbn* ^*−/−*^ SCIs, while the numbers in pro‐ and anti‐inflammatory cells were not altered. Oil Red O staining suggests that the abundance of IBA1 signal in the lesion core could originate from foam cells of macrophage origin, leading to increased inflammation possibly slowing the physiological tissue regeneration following SCI.

### 3.5 Increased Neuronal Degeneration in *Dbn* ^*−/−*^ SCI Animals

**FIGURE 6:**
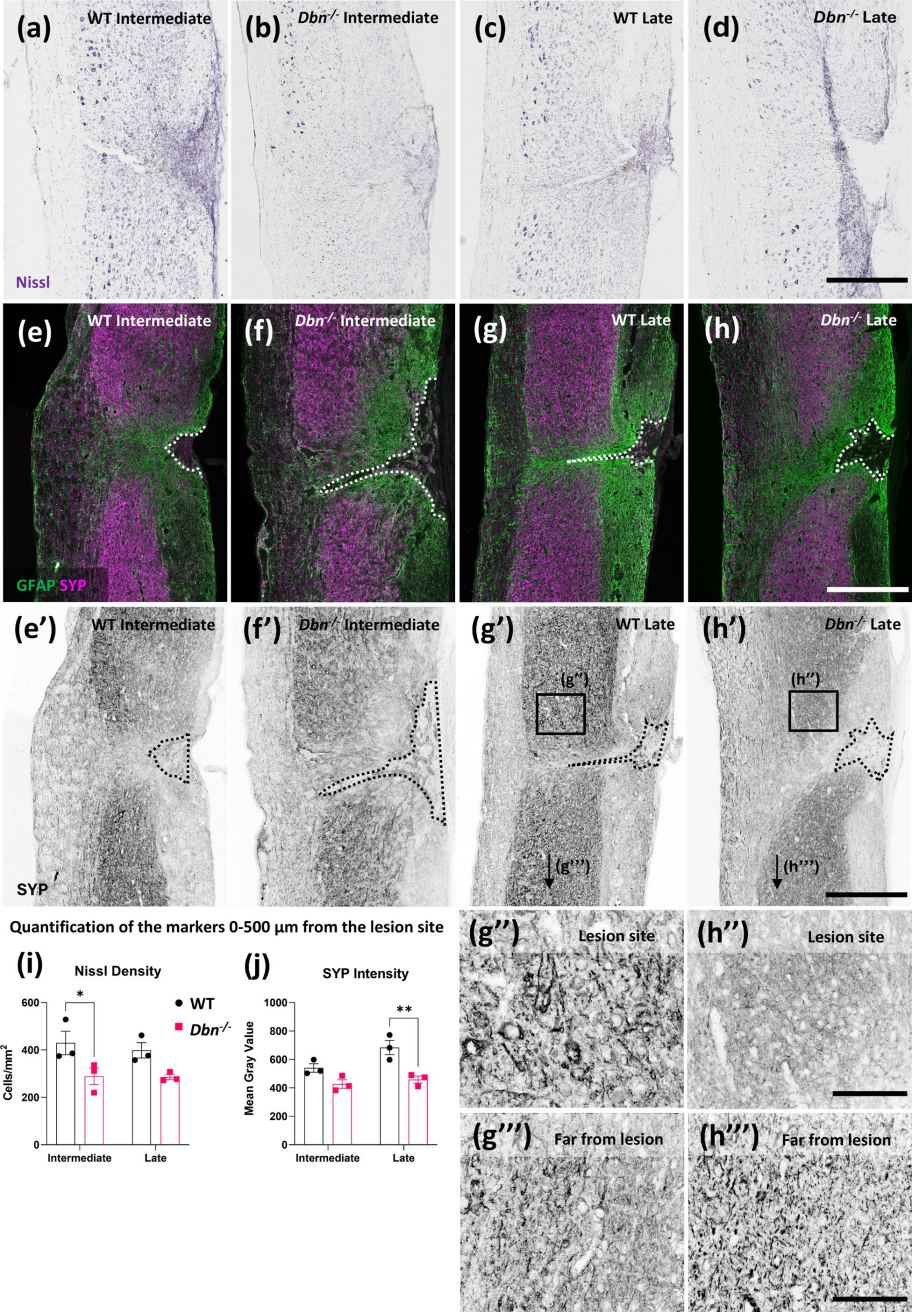
Increased neuronal degeneration in *Dbn* ^*−/−*^ animals following thoracic SCI**—**(a–d) Representative images of Nissl histology in WT (a, c) and *Dbn* ^*−/−*^ (b, d) tissue following SCI at intermediate and late timepoints, respectively, scale bar 500 μm. (e–h) Representative images of GFAP and synaptophysin (SYP) co‐labeling in WT (e, g) and *Dbn* ^*−/−*^ (f, h) tissue following SCI at intermediate and late timepoints, respectively, scale bar 500 μm. (e’–h’) SYP signal in grayscale corresponding to (e–h) respectively, scale bar 500 μm. (g”, h”) Magnifications of the SYP signal next to the lesion site, scale bar 100 μm. (g”’, h”’) Magnifications of the SYP signal far from the lesion site, scale bar 100 μm. (i) Density of Nissl bodies in gray matter adjacent to the lesion site (0–500 μm), (j) quantification of SYP signal intensity in gray matter adjacent to the lesion site (0–500 μm). Data shown as mean ± SEM, (*N* = 3), ns *p* ≥ 0.05, *p < 0.05, **p < 0.01, ***p < 0.001, ****p < 0.0001, two‐way ANOVA, Bonferroni‐Šídák's multiple comparison test.

We next studied potential changes in neuronal cell death in response to injury. We examined and quantified neuronal loss by using Nissl histology and quantified the density of Nissl bodies in the gray matter adjacent to the lesion site (0–500 μm from the lesion border, Figure 6a–d). At intermediate timepoints, the density of Nissl bodies is significantly reduced in *Dbn* ^*−/−*^ tissue compared to WT (Figure 6i). The reduction in Nissl bodies in *Dbn* ^*−/−*^ mice is related to the injury conditions, as the density of Nissl bodies was not statistically different in spinal cords of uninjured WT and *Dbn* ^*−/−*^ animals (Figure S6).

In addition to Nissl histology, we analyzed the abundance of synapses via immunohistochemical labeling of the presynaptic protein synaptophysin. In the spinal cord, synaptophysin is abundantly expressed in presynaptic terminals of neurons and is particularly prominent in the gray matter (Chung et al. 2019) Immediately following SCI, synaptophysin expression is reduced, consistent with the loss of synaptic connections due to neuronal damage and axonal degeneration (Masliah et al. 1991). Despite the initial loss, the spinal cord exhibits some degree of plasticity in response to injury. As part of the repair process, reactive synaptogenesis, the formation of new synaptic connections, occurs in the weeks after injury (O'Shea et al. 2017). To study neuronal plasticity at different time points after SCI, we immunolabeled synaptophysin at intermediate and late phases after SCI (Figure 6e–h; e’–h’). In both WT and *Dbn* ^*−/−*^ mice, synaptophysin intensity was decreased at the intermediate time point. However, at the late time point, WT mice showed an increased synaptophysin signal compared to *Dbn* ^*−/−*^ mice, indicating partial recovery of synaptic density in WT but not in *Dbn* ^*−/−*^ tissue. Confocal microscopy at higher magnification revealed more distinct and prominent synaptophysin‐positive structures in the WT in proximity to the lesion site (Figure 6g”) than in *Dbn* ^*−/−*^ (Figure 6h”). Details of SYP signal from tissue far from the lesion site show similar levels of signal intensity in the WT and *Dbn* ^*−/−*^ animals (Figure 6g”’, h”’). No statistically significant differences were observed in the signal intensity and distribution of the SYP signal in uninjured *Dbn* ^*−/−*^ tissue when compared to WT (Figure S6).

Changes in Nissl body density and synaptophysin intensity were prominent in the regions adjacent to the lesion core, whereas changes in neurodegeneration were not significant in more distant regions. These findings correlate with the tissue morphology observed with Luxol Fast Blue staining (Figure S7) where only mild demyelination was observed in *Dbn* ^*−/−*^ at the intermediate and late time points around the lesion core region. Areas distant from the lesion core showed similar levels of myelinization in both groups. This finding was confirmed by immunolabeling of the marker myelin associated glycoprotein (MAG). Immunohistochemical analyses of MAG showed similar patterns of myelinization in both groups. In addition, we could detect in both WT and *Dbn* ^*−/−*^ mice well defined myelin structures at the late timepoint post SCI. Therefore, the remyelination at later phase post SCI is not affected in *Dbn* ^*−/−*^ mice (Figure S7). In summary, we demonstrated an increased neurodegeneration and synaptic loss during the course of SCI in *Dbn* ^*−/−*^ mice while (re)myelination was not affected.

### 3.6 Impaired Astrocyte Polarization and Process Organization in *Dbn* ^*−/−*^ Mice Following SCI

**FIGURE 7:**
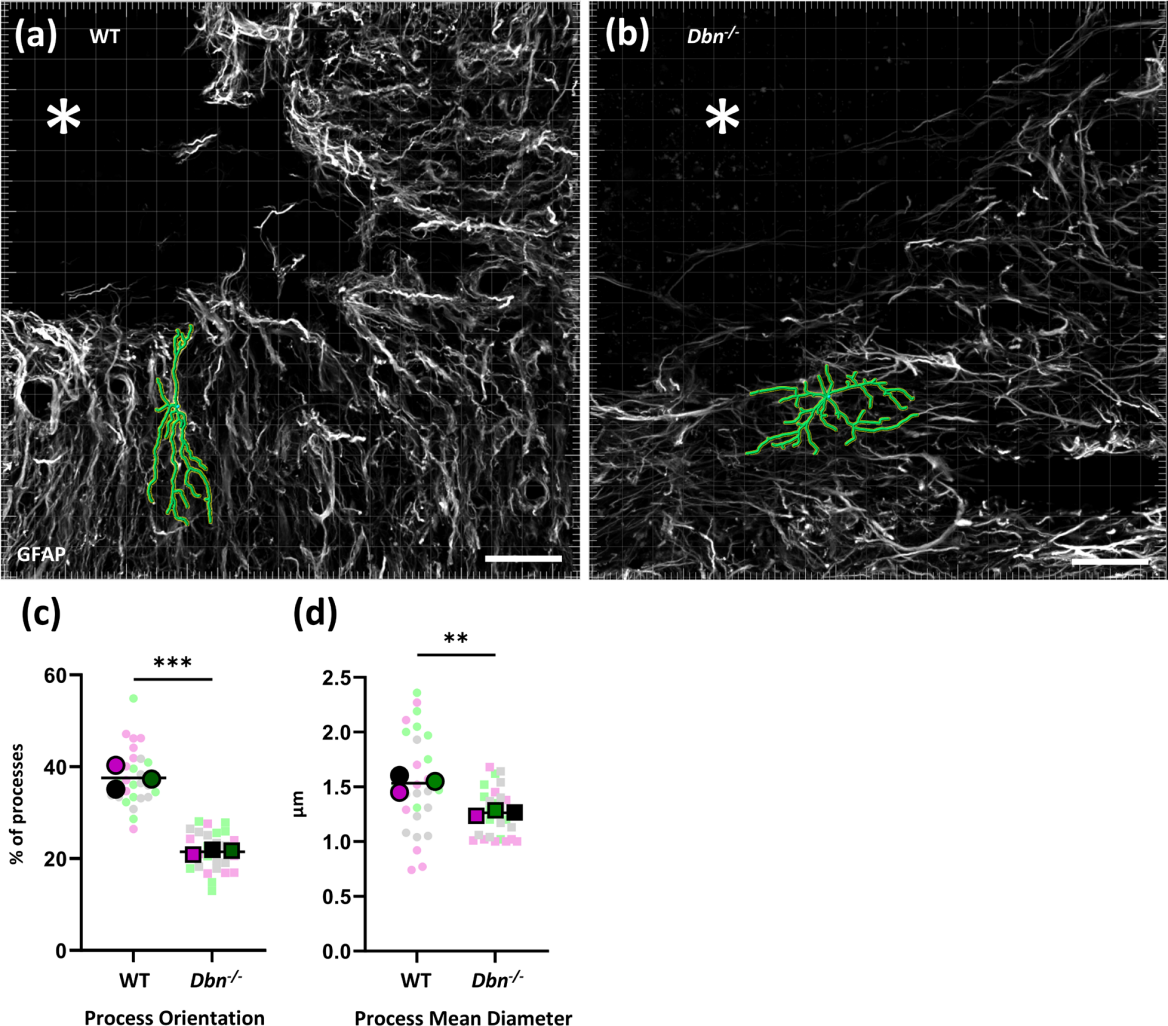
Loss of DBN in *Dbn* ^*−/−*^ mice affects polarization of astrocytes at the lesion site. (a, b) Reconstructions of GFAP signal from WT (a) and *Dbn* ^*−/−*^ (b) tissues. Overlay of the mask created in Imaris over the GFAP positive astrocytic network adjacent to the lesion site at the early timepoint shows representative cell (in green) used for analysis, asterisk indicates center of the lesion, scale bar 50 μm. (c, d) Graphs show quantifications of astrocyte morphometric measurements (c) percentage of astrocytic processes oriented towards lesion center. (d) Astrocyte process mean diameter. Data in superplots show overlay of individual values of analyzed cells and mean for each animal, (*N* = 3 animals per group, *n* = 9 cells per animal), ns *p* ≥ 0.05, *p < 0.05, **p < 0.01, ***p < 0.001, ****p < 0.0001, two‐way ANOVA, Bonferroni‐Šídák's multiple comparison test.

To generate a protective and injury‐limiting boundary, reactive astrocytes change their morphology by polarizing and outgrowing processes towards lesion sites (see for review Schiweck et al. 2018). We analyzed these hallmarks of astrocyte reactivity in WT and SCI sites using morphometric measurements. To quantify the deficient polarization of the astrocytes in *Dbn* ^*−/−*^ tissues, we created a representation of the astrocytic net using the Imaris filament tracer function. From this representation, individual cells were selected, and we analyzed the orientation of their GFAP+ processes, defined as the angle of processes relative to the lesion epicenter, as well as the mean diameter of these processes (Figure 7a–b). We found that a significantly smaller percentage of the processes was oriented towards the epicenter of the lesion in *Dbn* ^*−/−*^ compared to WT (Figure 7c) indicating the inability to form palisade scars in the *Dbn* ^*−/−*^. WT astrocytes form a more densely packed scar of parallel processes oriented towards the lesion epicenter. Astrocytes in the *Dbn* ^*−/−*^ tissue were more interspersed, with many processes perpendicular to the desired orientation angle. The mean diameter of processes was also significantly smaller in *Dbn* ^*−/−*^ compared to WT (Figure 7d) which correlates with the reduced hypertrophy and diminished GFAP signal observed in Figure 2. We also performed morphometric analyses on GFAP+ astrocytes in intact spinal cords of WT and *Dbn* ^*−/−*^ mice. Neither the Sholl analysis as a readout for astrocyte complexity nor measuring GFAP signal intensity revealed significant differences between non‐reactive astrocytes in WT and *Dbn* ^*−/−*^ mice in the intact spinal cord (Figure S8). Thus, Dbn loss affects specifically the morphological changes which reactive astrocytes undergo in response to traumatic injuries to contain tissue damage and inflammation.

## 4 Discussion

We have characterized the function of actin binding protein DBN in SCI. Our findings show that DBN controls astrocyte polarization, an integral process for the encapsulation of lesion sites during the acute SCI phase. *Dbn* ^*−/−*^ mice exhibit enlarged SCI lesions and abnormally increased immune cell infiltration and neurodegeneration in the surrounding parenchyma. Moreover, DBN loss delays regenerative processes and tissue remodeling towards the development of the chronic post‐SCI state.

### 4.1 DBN Is Upregulated in Reactive Astrocytes Upon SCI

In response to SCI, DBN was upregulated in reactive astrocytes localized near the SCI lesion core, while GFAP‐positive astrocytes further from the lesion did exhibit a lower increase of DBN, indicating a spatially restricted, injury‐triggered response. The DBN upregulation peaked 7 days post injury when astrocytes polarize and outgrow processes towards lesion sites (Figures 1 and S1, S2). These findings suggest that astrocytes require DBN in particular when they undergo comprehensive morphological changes. This hypothesis is supported in our experiments using *Dbn* ^*−/−*^ mice. DBN deficiency disrupted astrocyte reactivity and scar border formation, resulting in reduced GFAP expression and an incomplete, disorganized scar border. Lesion sites showed increased cavitation (lower solidity index) and fragmented borders compared to wild‐type tissue, suggesting inadequate encapsulation of the injury (Figures 2 and 3). The injury area extended primarily along the rostro‐caudal axis (Figure S4), reflecting disorganization and exacerbation of damage. These structural deficits were particularly pronounced in the early and intermediate post‐injury phases, underscoring DBN's importance in coordinating the initial astrocytic response and ensuring proper scar maturation. In contrast to our previous findings in traumatic brain injury, we did not observe a complete loss of GFAP expression in astrocytes in *Dbn* ^*−/−*^ tissues at later time points after SCI (Schiweck et al. 2021), potentially due to the overriding influence of extensive tissue damage, which may mitigate the suppressive effect of DBN loss on astrocyte reactivity.

The observed defects in lesion encapsulation and scar formation indicate a major role of astrocytes in the SCI phenotype of this mouse model, where *Dbn* is already deleted in the germline. The abnormal neurodegeneration in the pan *Dbn* ^*−/−*^ mice could be interpreted as increased vulnerability of DBN‐deficient neurons. However, previous analyses of mice with neuron‐specific DBN deletion showed no increased susceptibility of DBN‐deficient neurons to injuries. This study also revealed that neither cultured microglia nor IBA1+ cells in injured or uninjured brain tissue express DBN (Schiweck et al. 2021). While contributions from other cell types in our SCI analyses cannot be fully excluded, the disrupted astrocytic polarization and impaired injury containment suggest that the primary mechanism involves astrocytes.

### 4.2 DBN‐Deficient Mice Show an Impairment in Fine Motor Skills in the Early Phase Post SCI

The DCC model leads to complete disruption of axons within the lesion area, along with damage to the meninges and rupture of the dorsal spinal vein, causing bleeding into the forming core of the lesion and peripheral immune cell infiltration. The DCC model affects primarily the dorsal corticospinal tract (CST) and ascending sensory pathways, leading to defects in fine motor skills (Attwell et al. 2018). We found that in motor function behavioral tests performed at various time points post‐SCI, DBN deficiency correlated with larger spinal lesions and delayed recovery, but did not result in significant differences in overall motor skills compared to WT littermates, as assessed by the Basso Mouse Scale (Basso et al. 2006) and the maximum speed rotarod test. Both *Dbn* ^*−/−*^ and WT mice exhibited a similar recovery trajectory in gross locomotor skills, with gradual improvements over 8 weeks post‐SCI (Figure 4). This suggests that the absence of DBN and the consequential delayed anatomical recovery do not exacerbate large‐scale motor impairments, at least in terms of basic locomotion. In addition, based on the BMS scores, we conclude that the experimental injury resulted in complete CST transection and gross motor deficit expected with this level of injury (Hill et al. 2009). However, the ladder walking test, which focuses on fine motor control, revealed a temporary deficit in the *Dbn* ^*−/−*^ group, with a significantly lower Metz‐Whishaw score observed at 2 weeks post‐injury (Figure 4). The Metz‐Whishaw score is a very sensitive sensorimotor test requiring fine motor skills, coordination, and integration of the sensory input from the paw (Metz and Whishaw 2009; Riek‐Burchardt et al. 2004). The combination of CST transection from the dorsal column tract lesion with aggravated deterioration of dorsal sensory pathways (cuneate and gracile) might explain the more pronounced deficit in the *Dbn* ^*−/−*^ animals observed in week 2 of behavioral testing. No statistical differences were observed between groups of naïve animals; this is in agreement with other studies that found the *Dbn* ^*−/−*^ animals perform normally in the open field locomotor and rotarod test (Kajita et al. 2024).

These findings indicate that Drebrin deficiency primarily affects fine motor skills in the acute phase after SCI. *Dbn* ^*−/−*^ mice showed more frequent missteps and paw placement errors in the ladder rung walking, which can be expected in the injury model used. Observed behavioral outcomes are also in line with the gradually subsiding phenotype regarding altered scar formation observed in histology (Figure 3) and the injury spread in the rostro‐caudal axis observed (Figure S4). These findings suggest that while DBN deficiency contributes to early‐phase deficits in fine motor control following SCI, these impairments diminish over time, indicating delayed but not permanently compromised recovery.

### 4.3 DBN Is Required to Limit Immune Cell Infiltration Following SCI

DBN is required for astrocytes to limit immune cell infiltration after SCI, as its absence reduces astrocytes' restrictive barrier function and permits IBA1+ cell accumulation within the lesion (Okada et al. 2006). In this study, we showed that loss of DBN dampens the restrictive function of spinal cord reactive astrocytes after SCI and is associated with increased accumulation of IBA1+ cells in the lesion core, with no significant changes in signal intensity or coverage in spinal cord tissue distant from the injury (Figure 5). IBA1, a protein expressed in activated microglia and macrophages, serves as a crucial marker for assessing immune responses in the CNS (Ohsawa et al. 2004). The increased density of IBA1+ cells in the lesion core in *Dbn* ^*−/−*^ mice suggests a more pronounced recruitment of microglia and peripheral macrophages to the lesion site. In WT injured tissue, microglia and macrophages recruit and proliferate in the injury site immediately, with a maximum increase approximately 7 days after injury, and persist for several months (Beck et al. 2010). Recent work has highlighted a synergistic role of reactive astrocytes and activated microglia in containment and isolation of infiltrating peripheral immune cells at the lesion core during the early phase after SCI. Between 7 and 14 DPI, microglia accumulate mainly around the lesion core, where they directly contact GFAP‐positive astrocytes, forming the so‐called “microglial scar” and contributing to the isolation of peripheral immune cells inside the lesion core (Bellver‐Landete et al. 2019). No significant changes in IBA1 + coverage or intensity in tissue distant from the lesion were detected in *Dbn* ^*−/−*^ mice compared to WT mice, suggesting that DBN loss affects local immune activation without affecting the broader immune landscape. Augmented IBA1 immunoreactivity at lesion sites in *Dbn* ^*−/−*^ mice could be due to the role of DBN in maintaining and modulating effective scar formation and thus limiting the proliferation and spreading of peripheral immune components.

We can assume that the increase in IBA1 signal at the lesion site comes primarily from infiltrating peripheral macrophages and pericytes and only to a lesser extent from activated proliferative microglia (Bellver‐Landete et al. 2019). To distinguish the different cell types present in the lesion core, we performed Oil Red O staining for neutral lipids and triglycerides after SCI and identified foam cells arising as a result of extensive phagocytosis of myelin and cellular debris at the injury site. In *Dbn* ^*−/−*^ mice, the increased abundance of these foam cells, accompanied by a heightened IBA1 signal, suggests an intensified macrophage‐mediated phagocytic activity targeting myelin debris (Figure 5). This finding is consistent with previous research highlighting the role of foam cells in debris clearance and their accumulation at sites of injury (X. Wang et al. 2015).

Microglia and macrophages are capable of adopting different phenotypes depending on the environmental stimuli they encounter. These phenotypes can be broadly classified into pro‐inflammatory (M1) and anti‐inflammatory (M2) states (Paolicelli et al. 2022). Pro‐inflammatory M1 microglia and macrophages are typically characterized by inducible nitric oxide synthase (iNOS) expression, while anti‐inflammatory M2 cells express arginase‐1 (Arg1). Immunolabeling for M1 (iNOS) and M2 (Arg1) cells revealed no significant difference in M1/M2 cell abundance between WT and *Dbn* ^*−/−*^ mice in both the lesion core and adjacent areas (Figure 5). Interestingly, increased iNOS immunoreactivity was detected in *Dbn* ^*−/−*^ tissue distant from the lesion site. However, the morphology, size, and distribution of iNOS‐expressing cells far from the lesion might indicate the neuronal nature of these cells, rather than the expected populations of microglia or macrophages. Upon injury, the expression and activity of iNOS in neurons represent a source of sustained and elevated NO release potentially contributing to inflammation and neurodegeneration (Heneka and Feinstein 2001). This activation was absent in uninjured *Dbn* ^*−/−*^ tissues (Figure S5), suggesting that the increased iNOS expression in neural tissue distant from the lesion site might be due to increased injury severity or increased cytotoxic agents' spread from the injured area.

### 4.4 DBN Promotes Neuronal Survival During SCI

Our previous study identified a substantial neuronal death in proximity to stab wound lesions in *Dbn* ^*−/−*^, but not in WT brains, with significant loss of NeuN‐positive and Nissl‐positive cell bodies starting from 7 days post injury (Schiweck et al. 2021). These findings indicate that loss of DBN leads to neurodegeneration upon brain stab injuries. Similarly, the significant increase in neuronal cell death and synaptic loss in *Dbn* ^*−/−*^ mice following SCI confirms our previous identification of a key role of DBN in neuroprotection and synaptic repair (Schiweck et al. 2021). In particular, 4 weeks post injury, Nissl staining revealed a decreased number of Nissl bodies in the gray matter adjacent to the lesion site in *Dbn* ^*−/−*^ mice compared to WT mice (Figure 6i). No significant differences in Nissl bodies were observable in uninjured spinal cords (Figure S6). This finding is in line with previous work showing no gross differences in neurons and tissue architecture in the uninjured brains of *Dbn* ^*−/−*^ mice (Willmes et al. 2017). In addition, we examined neurodegeneration by immunolabeling synaptophysin, an abundant presynaptic vesicle protein reflecting the synaptic density in the spinal cord (Janz et al. 1999). In both *Dbn* ^*−/−*^ and WT tissue, synaptophysin levels were initially reduced after SCI, consistent with loss of synaptic function due to axonal degeneration and neuronal damage (Figure 6). However, at the late time points, WT mice exhibited a partial recovery of synaptophysin expression, as revealed by distinct and prominent synaptophysin‐positive structures surrounding the lesion, suggesting some degree of reactive synaptogenesis, a regenerative process in which new synaptic connections are formed in response to injury (Masliah et al. 1991). This recovery of synaptophysin expression was significantly impaired in *Dbn* ^*−/−*^ mice, suggesting that defective scarring in the absence of DBN might cause a reduction or delay of repair mechanisms after SCI, in proximity to the lesion. At the intermediate and late time points post injury, *Dbn* ^*−/−*^ tissue only showed a mild demyelination, confined primarily to areas adjacent to the lesion core (Figure S7), while similar levels of myelination were detected in both genotypes far from the lesion area, suggesting that DBN loss primarily affects the directly injured tissue. These results are consistent with prior studies showing that demyelination tends to be localized to the lesion core after SCI, whereas distant areas of the spinal cord are relatively spared (Crowley et al. 2019).

### 4.5 DBN Regulates Astrocyte Polarization in Response to SCI

Astrocytes typically respond to CNS injury with hypertrophy, while cells in close proximity to the injury polarize and extend processes towards the lesion core (Schiweck et al. 2021). When we performed morphometric analysis to study astrocytes polarization at lesion areas, we revealed a significant reduction in GFAP‐positive astrocyte process numbers in the SCI astrocyte boundaries of *Dbn* ^*−/−*^ mice. Further analyses showed that in *Dbn* ^*−/−*^ mice only a smaller percentage of reactive astrocytes processes were oriented towards the lesion epicenter compared to WT animals. These findings demonstrate the inability of astrocytes in *Dbn* ^*−/−*^ mice to form palisade‐like structures to encapsulate SCI lesions (Figure 7). These findings are in line with the overall enlarged injury sites in *Dbn* ^*−/−*^ mice (Figure 3 and S4). Moreover, this phenotype is consistent with astrocyte polarization and outgrowth defects observed in mild traumatic brain injuries (Schiweck et al. 2021). No significant changes in astrocyte branching complexity were observed in uninjured tissue (Figure S8). The absence of morphological alterations in non‐reactive astrocytes in *Dbn* ^*−/−*^ mice supports our hypothesis that DBN specifically facilitates the morphological changes of reactive astrocytes in response to CNS injuries.

## 5 Conclusion

In conclusion, the inability of astrocytes in *Dbn* ^*−/−*^ tissues to form a dense, organized glial scar following SCI represents a significant disruption to the central nervous system's protective response. While glial scars are often viewed as barriers to axonal regrowth, their role in containing the lesion and limiting secondary damage is crucial during the early phases of SCI. The failure of astrocytes in *Dbn* ^*−/−*^ tissue to properly polarize and form these protective barriers likely contributes to the increased lesion size and neurodegeneration observed in these mice. These findings underscore the essential function of DBN in regulating astrocyte reactivity and astrocyte scar formation. Future research should focus on unraveling the molecular pathways through which DBN influences astrocytic behavior, as this could open new avenues for therapeutic strategies aimed at modulating glial scarring and enhancing recovery after CNS injury.

## Author Contributions

B.S. and M.O. contributed equally. B.S., M.O., K.Š., J.S., S.M., and R.H. performed the experiments. B.S., M.O., and K.Š. analysed the experiments. P.J., B.J.E., K.M., J.F., J.C.F.K., L.M.U., and P.B.S. supervised the experiments. P.J., B.J.E., J.F., L.M.U. and K.M. designed the study.

## Ethics Statement

All procedures were approved by the Ethics Committee of the Institute of Experimental Medicine of the ASCR and were performed in accordance with Act No. 77/2004 of the Czech Republic (ethics approval number: AVCR 7848/2022 SOV II).

## Conflicts of Interest

The authors declare no conflicts of interest.

## Supporting information


Figure S1.

Figure S2.

Figure S3.

Figure S4.

Figure S5.

Figure S6.

Figure S7.

Figure S8.

## Data Availability

The data that support the findings of this study are openly available in Dataset for Article: Drebrin upregulation regulates astrocyt at https://zenodo.org/uploads/14006017, reference number https://doi.org/10.5281/zenodo.14006017.
