# Supplementary figures and images for: Drebrin Upregulation Regulates Astrocyte Polarization and Supports Tissue Recovery After Spinal Cord Injury in Mice
Source: Glia. 2025 Jun 11;73(9):1910–24. doi: 10.1002/glia.70048 (PMC12313004; doi:10.1002/glia.70048)

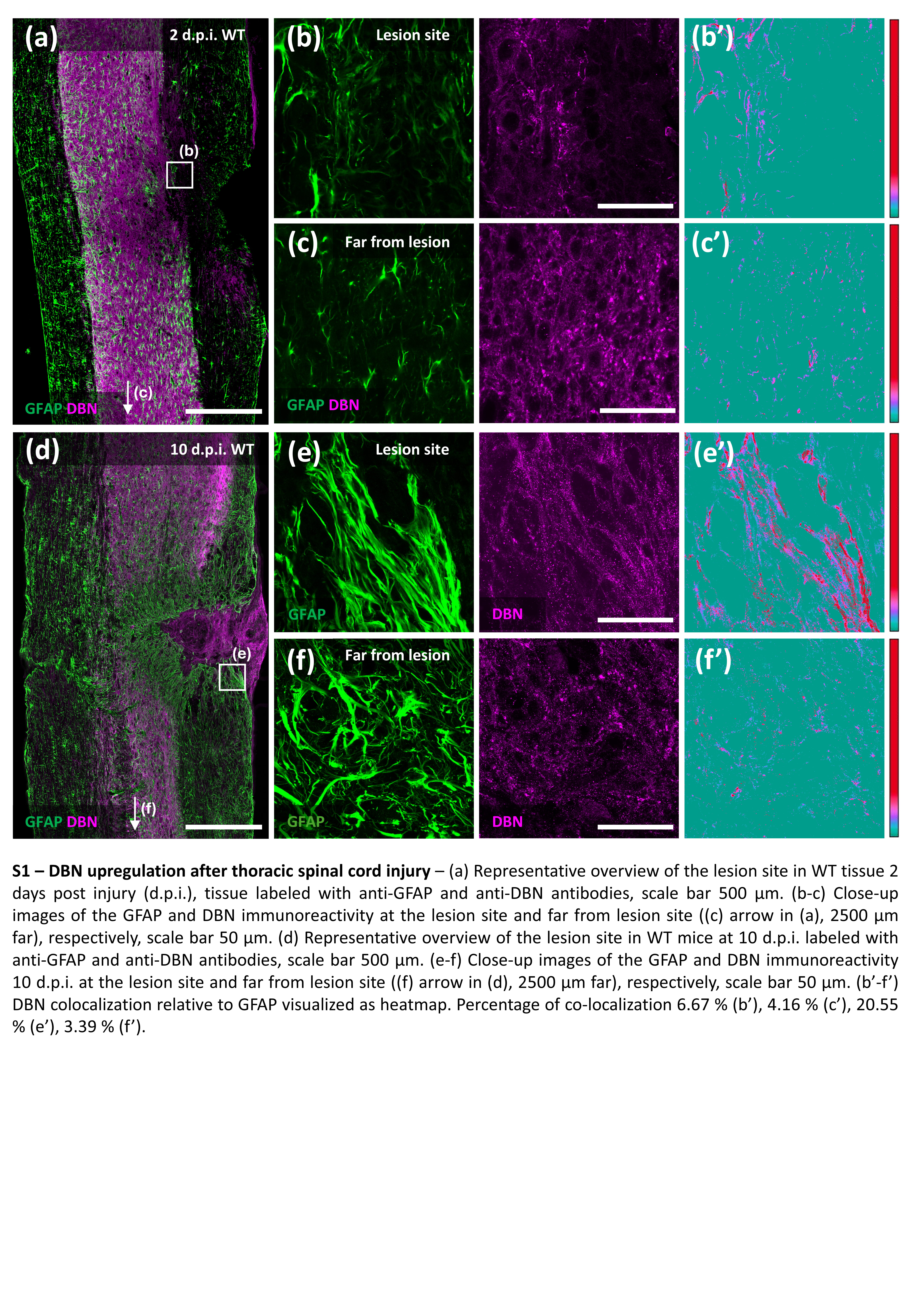

Supplement: Supplementary file 1 — Figure S1. [file GLIA-73-1910-s006.tif]

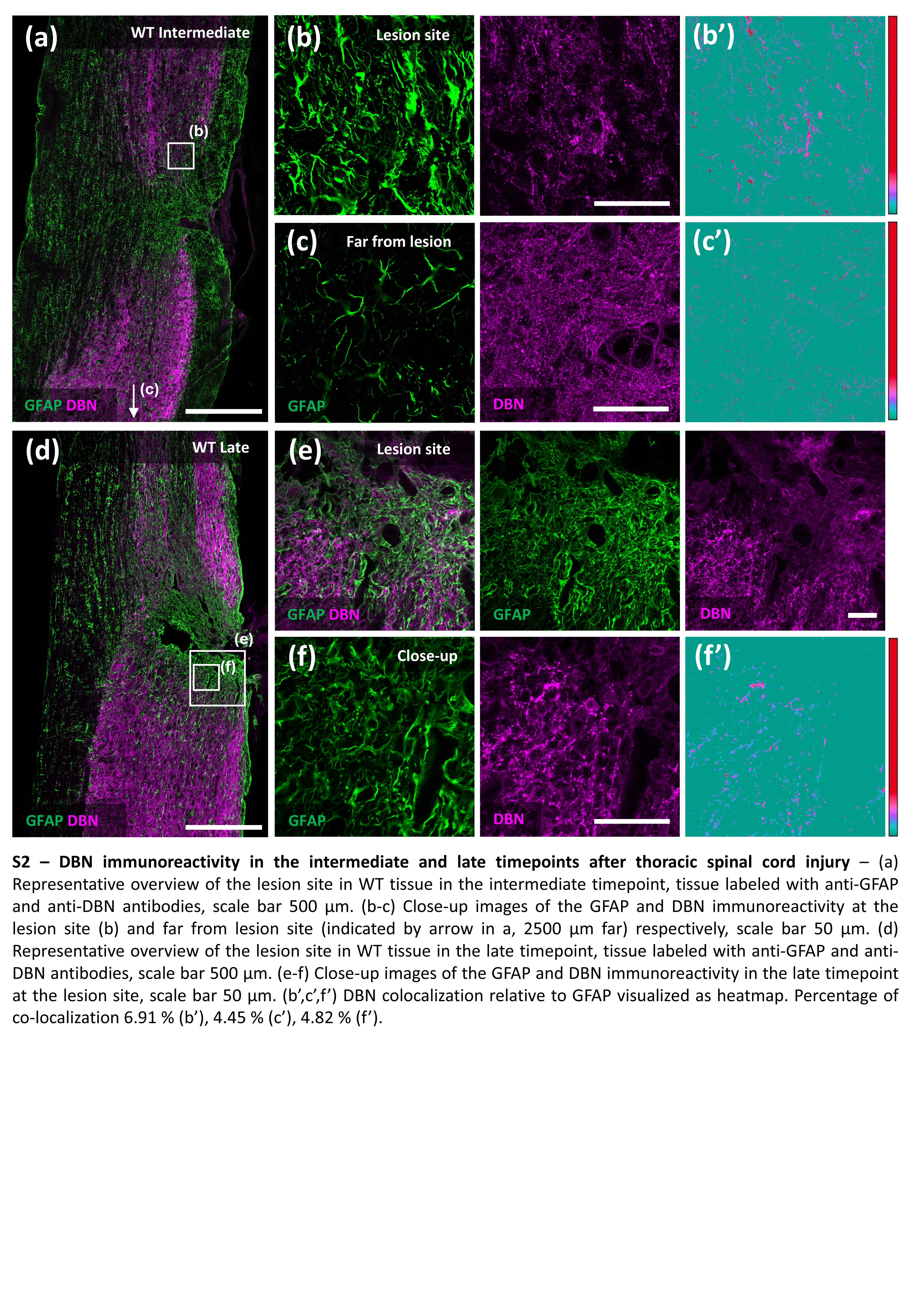

Supplement: Supplementary file 2 — Figure S2. [file GLIA-73-1910-s001.tif]

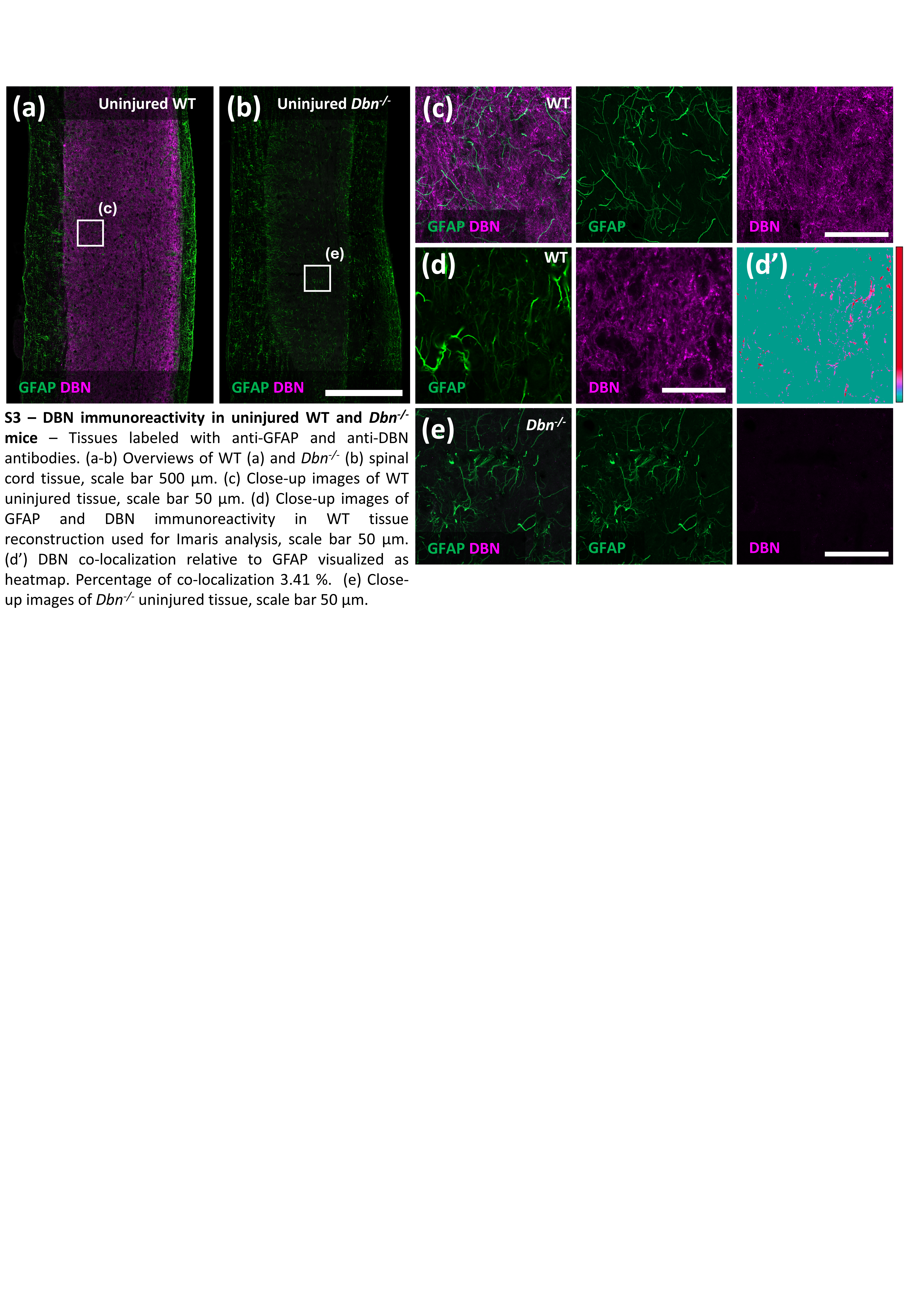

Supplement: Supplementary file 3 — Figure S3. [file GLIA-73-1910-s005.tif]

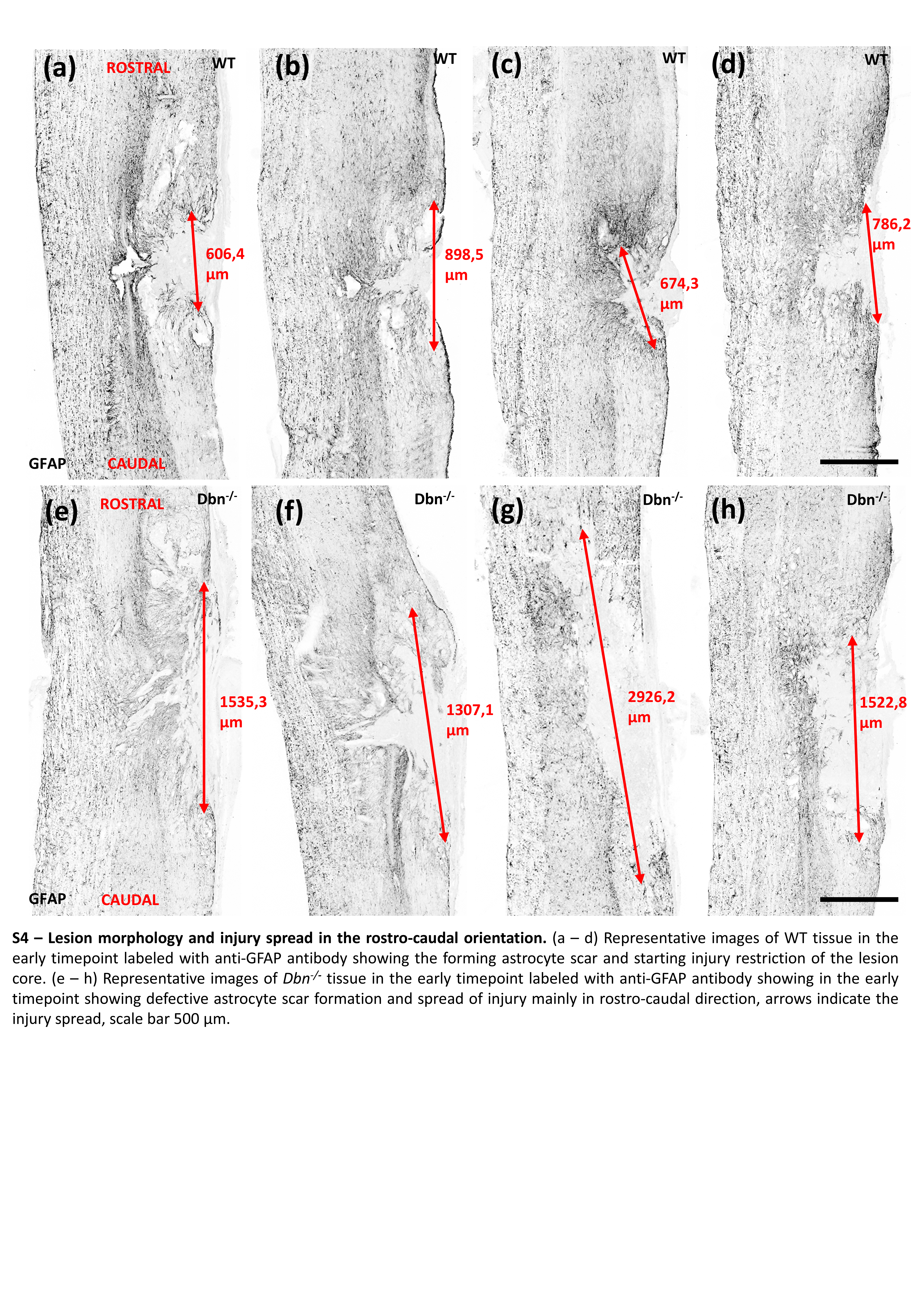

Supplement: Supplementary file 4 — Figure S4. [file GLIA-73-1910-s004.tif]

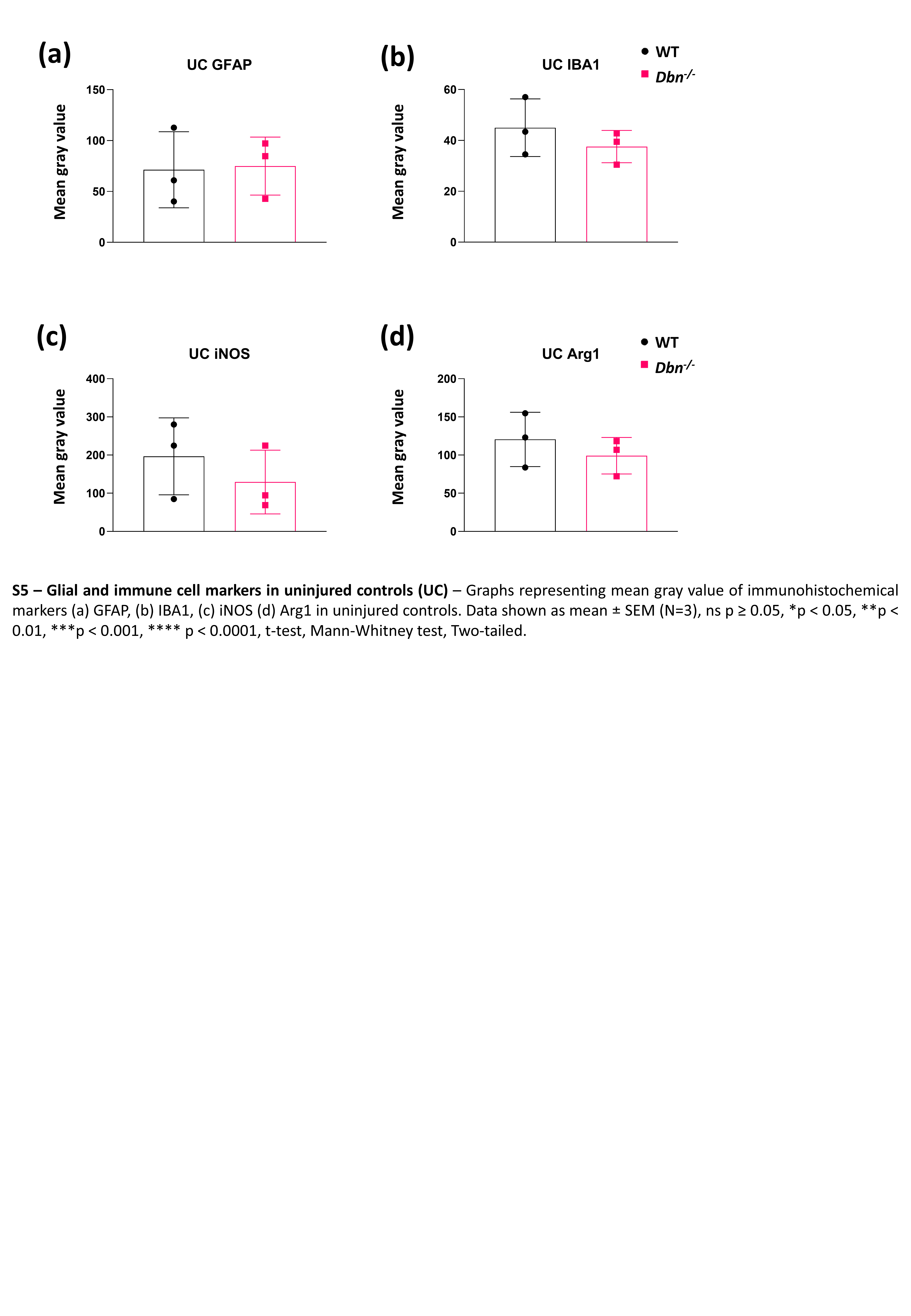

Supplement: Supplementary file 5 — Figure S5. [file GLIA-73-1910-s002.tif]

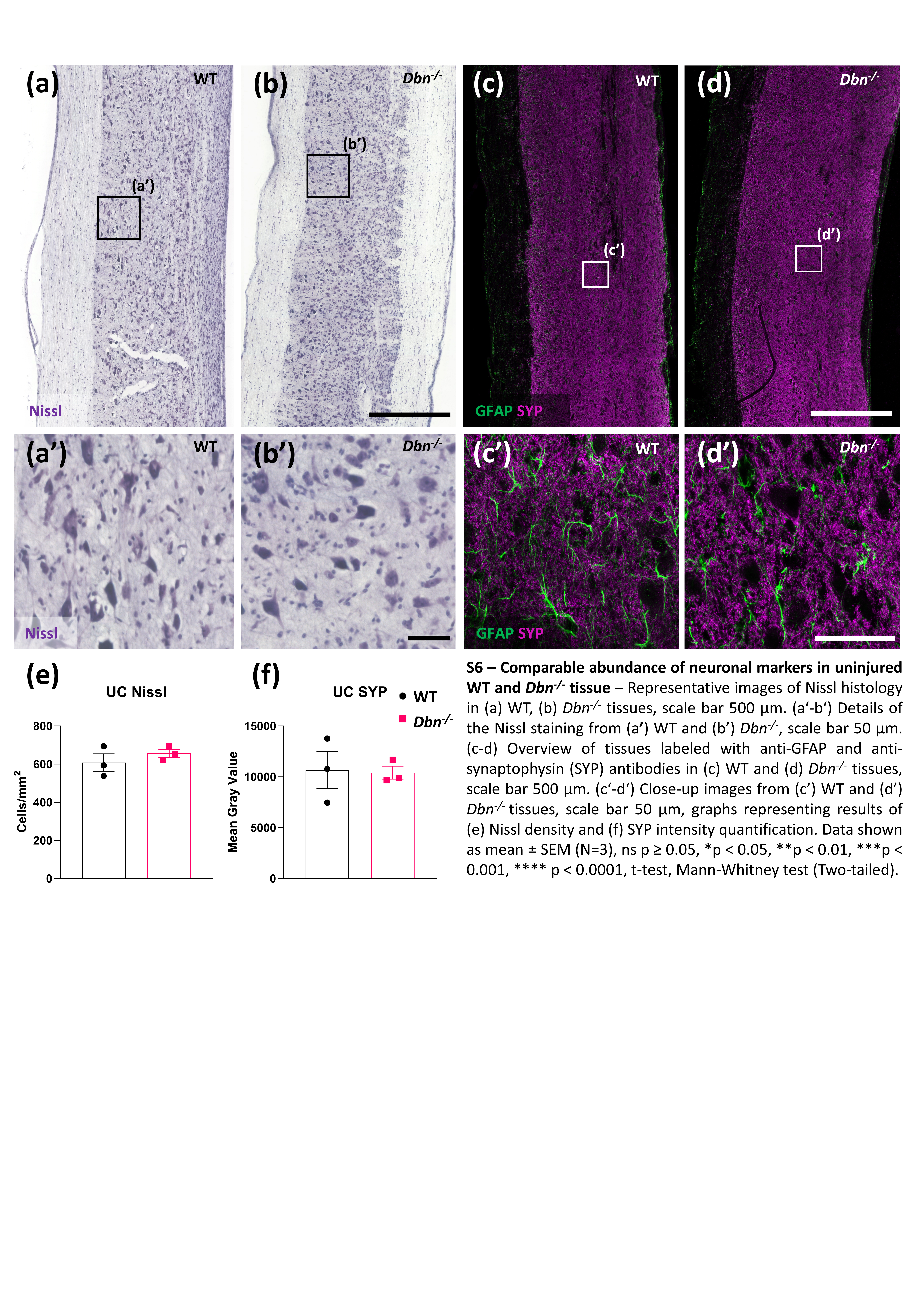

Supplement: Supplementary file 6 — Figure S6. [file GLIA-73-1910-s007.tif]

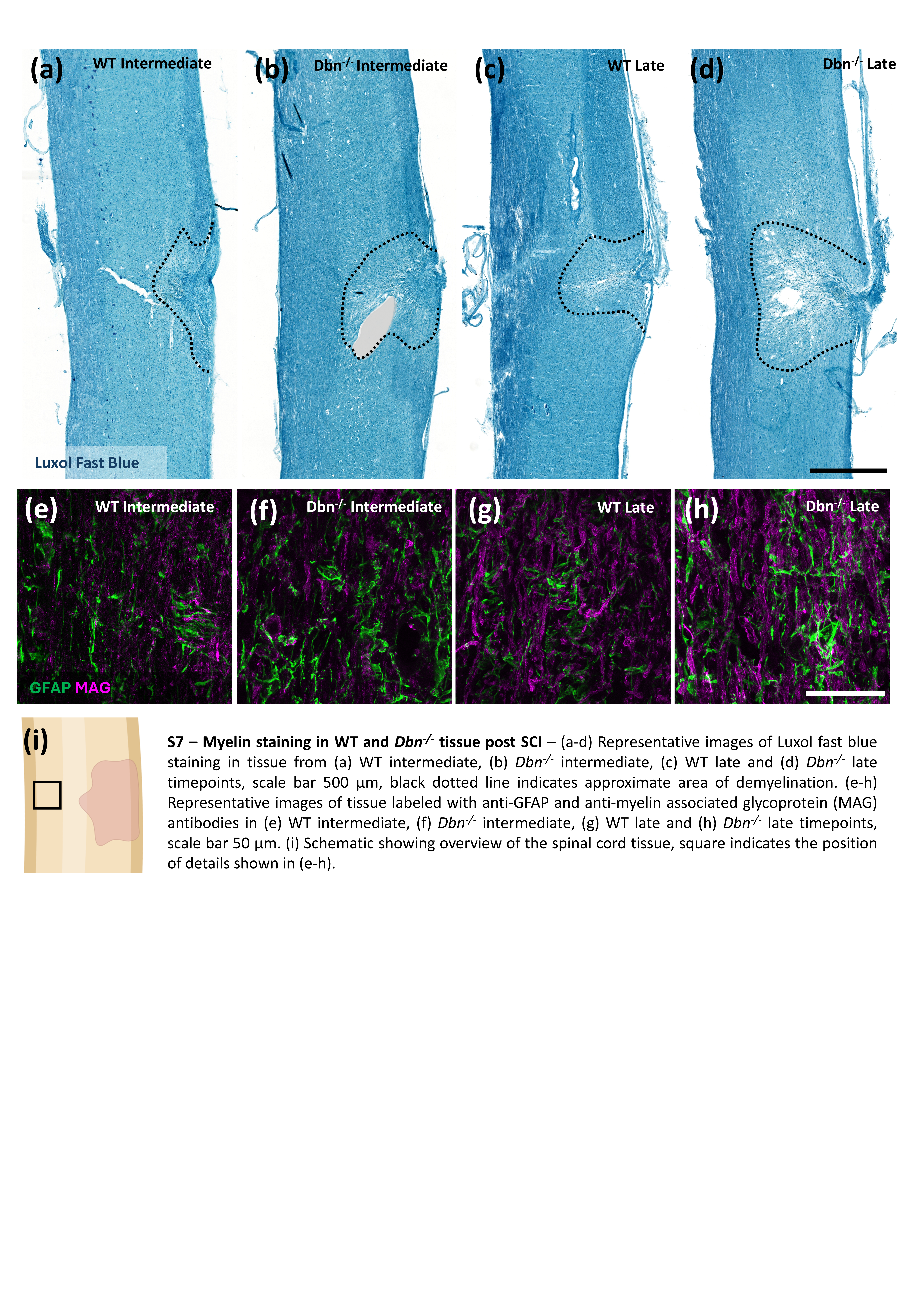

Supplement: Supplementary file 7 — Figure S7. [file GLIA-73-1910-s008.tif]

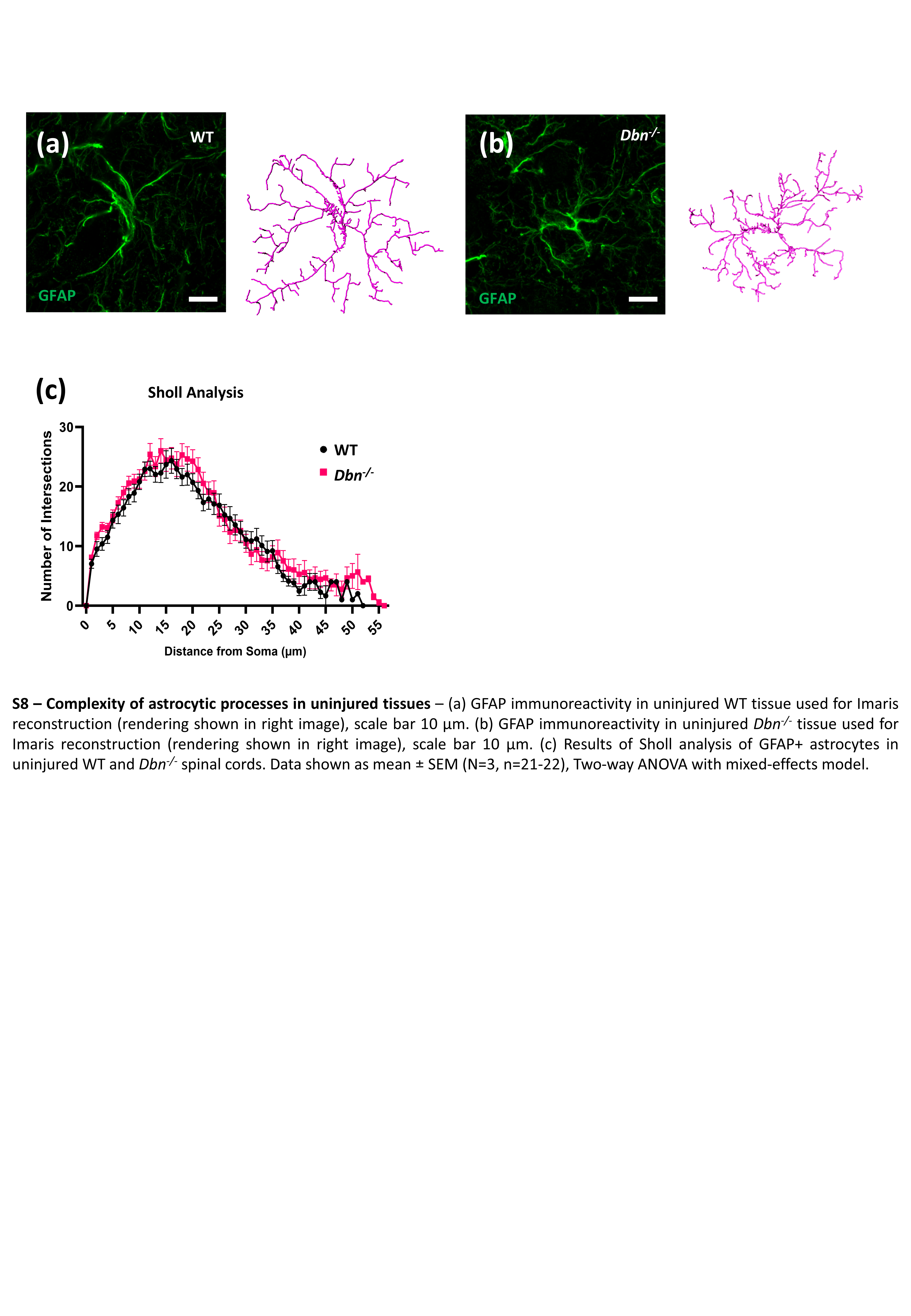

Supplement: Supplementary file 8 — Figure S8. [file GLIA-73-1910-s003.tif]
